# The FLRT3-UNC5B checkpoint pathway inhibits T cell–based cancer immunotherapies

**DOI:** 10.1126/sciadv.adj4698

**Published:** 2024-03-01

**Authors:** Kushal Prajapati, Chuan Yan, Qiqi Yang, Steven Arbitman, Daniel P. Fitzgerald, Sasan Sharee, Jahangheer Shaik, Jason Bosiacki, Kayla Myers, Ana Paucarmayta, Dorothy M. Johnson, Thomas O’Neill, Subhadip Kundu, Zachary Cusumano, Solomon Langermann, David M. Langenau, Shashank Patel, Dallas B. Flies

**Affiliations:** ^1^ NextCure Inc., Beltsville, MD 20705, USA.; ^2^ Molecular Pathology and Cancer Center, Massachusetts General Hospital Research Institute, Charlestown, MA 02129, USA.; ^3^ Harvard Stem Cell Institute, Cambridge, MA 02139, USA.

## Abstract

Cancers exploit coinhibitory receptors on T cells to escape tumor immunity, and targeting such mechanisms has shown remarkable clinical benefit, but in a limited subset of patients. We hypothesized that cancer cells mimic noncanonical mechanisms of early development such as axon guidance pathways to evade T cell immunity. Using gain-of-function genetic screens, we profiled axon guidance proteins on human T cells and their cognate ligands and identified fibronectin leucine-rich transmembrane protein 3 (FLRT3) as a ligand that inhibits T cell activity. We demonstrated that FLRT3 inhibits T cells through UNC5B, an axon guidance receptor that is up-regulated on activated human T cells. FLRT3 expressed in human cancers favored tumor growth and inhibited CAR-T and BiTE + T cell killing and infiltration in humanized cancer models. An FLRT3 monoclonal antibody that blocked FLRT3-UNC5B interactions reversed these effects in an immune-dependent manner. This study supports the concept that axon guidance proteins mimic T cell checkpoints and can be targeted for cancer immunotherapy.

## Teaser

FLRT3-UNC5B is an immune coinhibitory pathway that can be targeted to enhance T cell–based therapies.

## INTRODUCTION

Cancer immunotherapy, one of the key scientific breakthroughs against cancer, was pioneered by targeting T cell coinhibitory immune checkpoint inhibitors (ICIs), with U.S. Food and Drug Administration–approved therapies including anti-CTLA-4 (ipilimumab) and anti–PD-1 (nivolumab) for melanoma in 2011 and 2014, respectively (*1*). Since then additional ICIs have been identified and developed for the clinic (*2*–*5*). Despite outstanding efficacy in several cancers, most patients remain nonresponsive to current ICI immunotherapies (*6*). This suggests that many additional and unknown immune inhibitory mechanisms may be active or adaptively activated in cancer, leading to evasion of T cell immunity, which remain to be fully explored.

Axon guidance molecules (AGMs) are a family of proteins that play a pivotal role in neuronal development by providing molecular cues to guide the migration of developing neurons (*7*). Numerous studies have shown that the role of AGMs such as semaphorins, ephrins, slits, and netrins go beyond neuronal development to cell migration, adhesion, and apoptosis (*8*) as well as tumorigenesis, angiogenesis, and metastasis in cancers (*9*–*15*). Recently, the ability of AGMs to regulate inflammation and immune response in and outside of the neuronal system has received attention in the field (*16*, *17*). Here, we present compelling evidence that AGM ligand-receptor pairs mimic coinhibitory pathways that can disrupt antitumor T cell immunity.

## RESULTS

### Discovery screening was performed to identify axon guidance coinhibitory mimics

AGMs were identified through queries made in publication, gene, transcript, and protein databases including PubMed, National Center for Biotechnology Information (NCBI), and Ensembl. The relationship of compiled AGMs was characterized phylogenetically (fig. S1 and table S1). Evaluation of the axon guidance gene set using The Cancer Genome Atlas (TCGA) database confirmed that many are expressed in cancer (fig. S2).

**Fig. 1.**
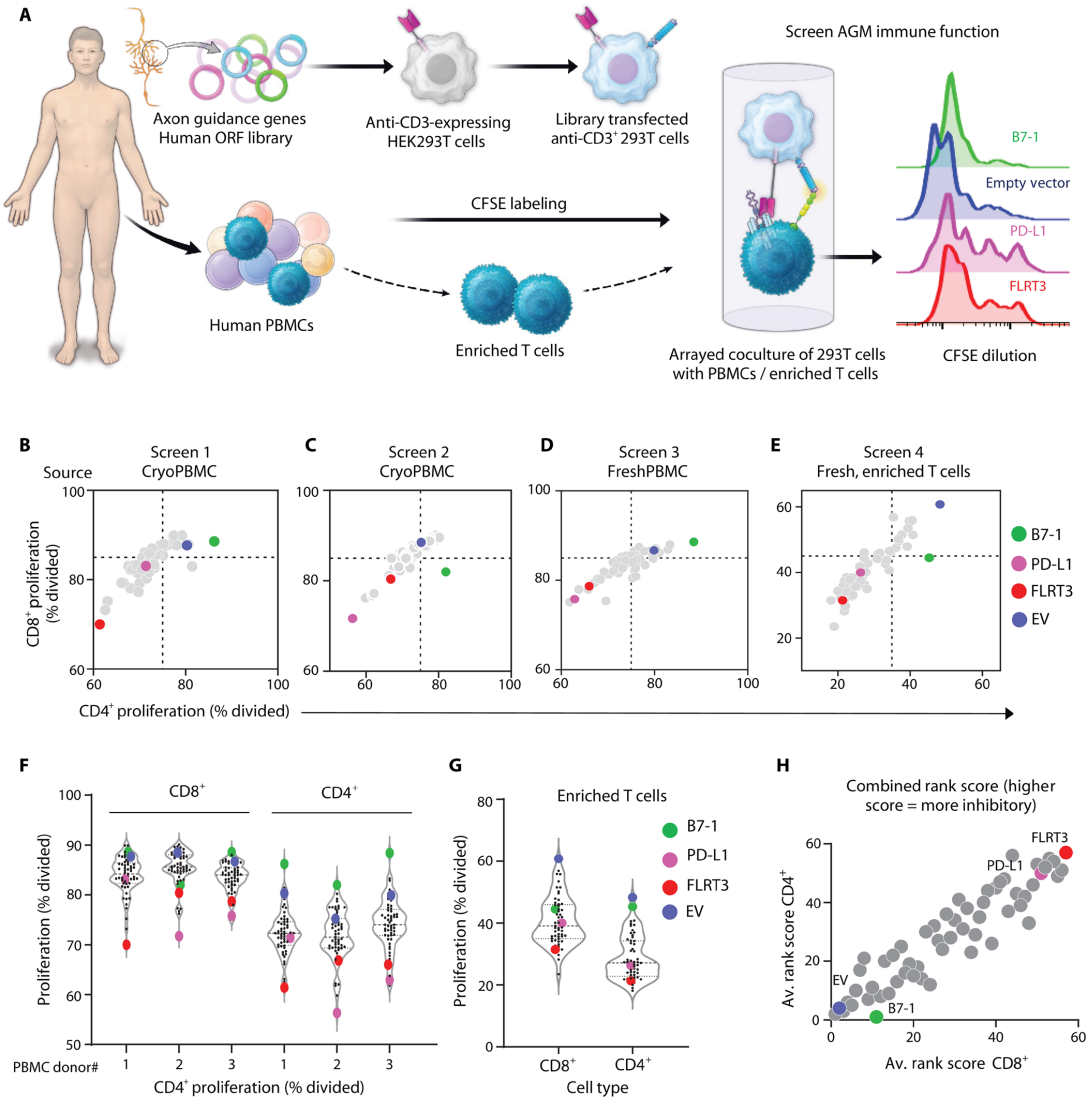
An axon guidance coinhibitory mimicry screen using primary human T cells. (**A**) A subset of human AGMs was screened by cell-cell interaction-based gain of function for T cell proliferation. (**B** to **E**) CD8^+^ and CD4^+^ T cell proliferation based on CFSE dilution from total cryopreserved PBMCs (B and C), fresh PBMCs (D), or fresh enriched T cells (E) from healthy donor leukopaks. (**F** and **G**) Violin plots of CD8^+^ and CD4^+^ T cell proliferation from total PBMC screens (F) and enriched T cell screen (G). Each data point in (B) to (G) represents one technical replicate for each donor. Data from single screens performed over four different donors are shown. (**H**) All genes were rank-scored for individual screens for their effect on the T cell proliferation. The averaged rank scores for all screens are shown, with the most costimulatory and inhibitory scores being 1 and 57, respectively, to identify candidate immune inhibitory genes.

Out of 177 axon guidance genes, we initially generated an open reading frame (ORF) library of 54 AGMs to determine their role in immune modulation in cancer. To this end, we established a gain-of-function genetic screen to identify AGMs that, when expressed as ligands, could interact with their cognate receptors on T cells to coinhibit or costimulate CD3ζ-based signaling on primary T cells. To achieve this, we first generated 293T cells that stably expressed an anti-CD3 (OKT3) single-chain variable fragment (scFv) tethered to the membrane (293T-OKT3). We transfected 293T-OKT3 cells with the AGM ORF library in arrayed format and cocultured them with peripheral blood mononuclear cells (PBMCs) or isolated T cells to measure the effect of individual AGM on T cell proliferation, a surrogate measure of T cell activation (Fig. 1A). We performed four independent screens using freshly isolated or cryopreserved PBMCs or enriched T cells sourced from different donors since to evaluate molecules that had similar, potentially robust effects on T cells in variable conditions. B7-1 and PD-L1 were included as T cell costimulatory and coinhibitory controls, respectively. In most cases B7-1 added little costimulation benefit due to strong stimulation by OKT3 expressed on 293T cells (Fig. 1). Similarly, the empty vector control heightened OKT3 stimulation, particularly for CD8^+^ T cells and enriched T cells. As such, AGMs that inhibited T cell under strong stimulatory conditions were considered for further validation.

Several AGMs that inhibited T cell proliferation comparable to or better than PD-L1 were identified, including ephrins (EFNA2, EFNB1, EPHA1), plexins (including PLXNB3), and semaphorins (including SEMA7A) (table S1). Upon analyzing consistency of inhibitory effects on both CD8^+^ and CD4^+^ T cell proliferation across four different screens, fibronectin leucine-rich transmembrane 3 (FLRT3) was identified as T cell inhibitor with activity analogous to PD-L1 as a measure of T cell inhibition (Fig. 1, A to H). Other T cell regulators that remain of interest for further evaluation include several ephrins (EFNA2, EFNB1, EPHA1), plexins (including PLXNB3), and semaphorins (including SEMA7A) (table S1). To identify the top costimulatory and coinhibitory AGMs, we equated rank-score metric for immune inhibitory effect of all the AGMs across all the screening datasets. FLRT3 was identified as the most inhibitory AGM for further validation (Fig. 1H).

### Validation of FLRT3 was validated as a T cell checkpoint inhibitor

FLRT3 is a leucine-rich repeat (LRR)–containing type I transmembrane protein (*18*) that functions as a repulsive axon guidance factor during development (*18*–*25*). While some recent studies implicated FLRT3 in mediating tumor immune escape in acute myeloid leukemia and breast cancer (*26*, *27*), its role in directly modulating T cell activity in cancer has not been explored.

**Fig. 2.**
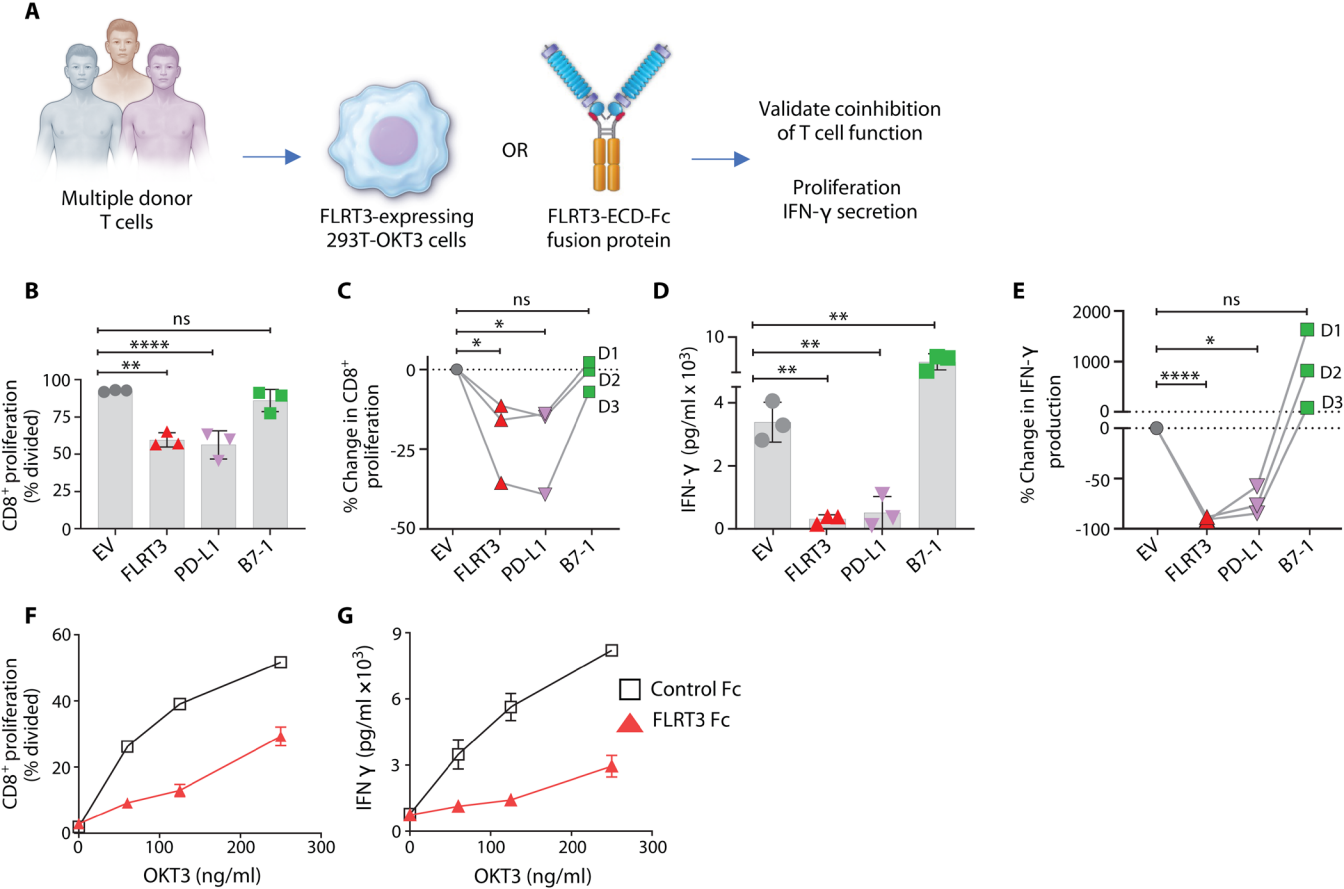
FLRT3 inhibits T cell proliferation and function in vitro. (**A**) Illustration of experimental approaches for validation of FLRT3 screening results using 293T-OKT3 cell-based or FLRT3-Fc fusion protein-based systems. (**B** to **E**) CFSE-labeled human PBMCs from three healthy donors were cocultured with 293T-OKT3 cells transfected with FLRT3, PD-L1, and B7-1 genes followed by analysis of proliferation by CFSE dilution (B and C) and IFN-γ production by ELISA (D and E). Quantification of proliferating CD8^+^ T cells (B) and IFN-γ production (D) is shown for one representative donor. Each data point represents one technical replicate, and error bars denote SD. (C and E) Percent change mediated by individual genes in CD8^+^ T cell proliferation (C) and IFN-γ production (E) in comparison to EV control for all three donors in a pairwise fashion. (**F** and **G**) Human PBMCs from a healthy donor were labeled with CFSE and activated on plates coated with titrated OKT3 and FLRT3-Fc (10 μg/ml). Proliferation of CD8^+^ T cells was analyzed by CFSE dilution via flow cytometry (F), and IFN-γ production was measured in the supernatants by ELISA (G). Error bars denote SD. *n* = 3 technical replicates for each data point. Data representative of at least two independent experiments. Statistical significance was determined for (B) and (D) by one-way ANOVA with Tukey’s post hoc test for multiple comparisons and for (C) and (E) by repeated-measures one-way ANOVA with Tukey’s post hoc test for multiple comparisons to account for matched values for individual donors. For all the data, *P < 0.05, **P < 0.01, ***P < 0.001, ****P < 0.0001.

To validate screen results, we assessed FLRT3 function via independent methods using T cells from additional human donors (Fig. 2A, illustration). Analogous to gene library screening, we evaluated the effect of FLRT3-expressed 293T-OKT3 cells on T cell proliferation, as well as interferon-γ (IFN-γ) production. We included B7-1– and PD-L1–expressing 293T-OKT3 cells as costimulatory and coinhibitory controls, respectively. FLRT3 inhibited T cell proliferation (Fig. 2, B and C) and suppressed IFN-γ production similar to PD-L1 across all donors (Fig. 2, D and E). To rule out direct effects of FLRT3 transfection on 293T-OKT3, we confirmed that FLRT3 did not affect cell growth or viability (fig. S3, A and B). While we observed slightly increased FLRT3 inhibition in CD4^+^ versus CD8^+^ T cell proliferation, this difference was not statistically significant (Fig. 2, B and C, and fig. S3, C and D).

A fusion protein consisting of extracellular domain of FLRT3 attached to human IgG1 Fc backbone (FLRT3 Fc) was used to test the direct effect of FLRT3 binding to human T cells. FLRT3 Fc inhibited CD8^+^ T cell proliferation and IFN-γ production in the presence of titrated concentrations of plate-coated OKT3 (Fig. 2, F and G). These data validated gene library screening results and supported FLRT3 as a T cell coinhibitory AGM with a direct coinhibitory effect on T cells.

### FLRT3 checkpoint function was mediated through the UNC5B receptor

**Fig. 3.**
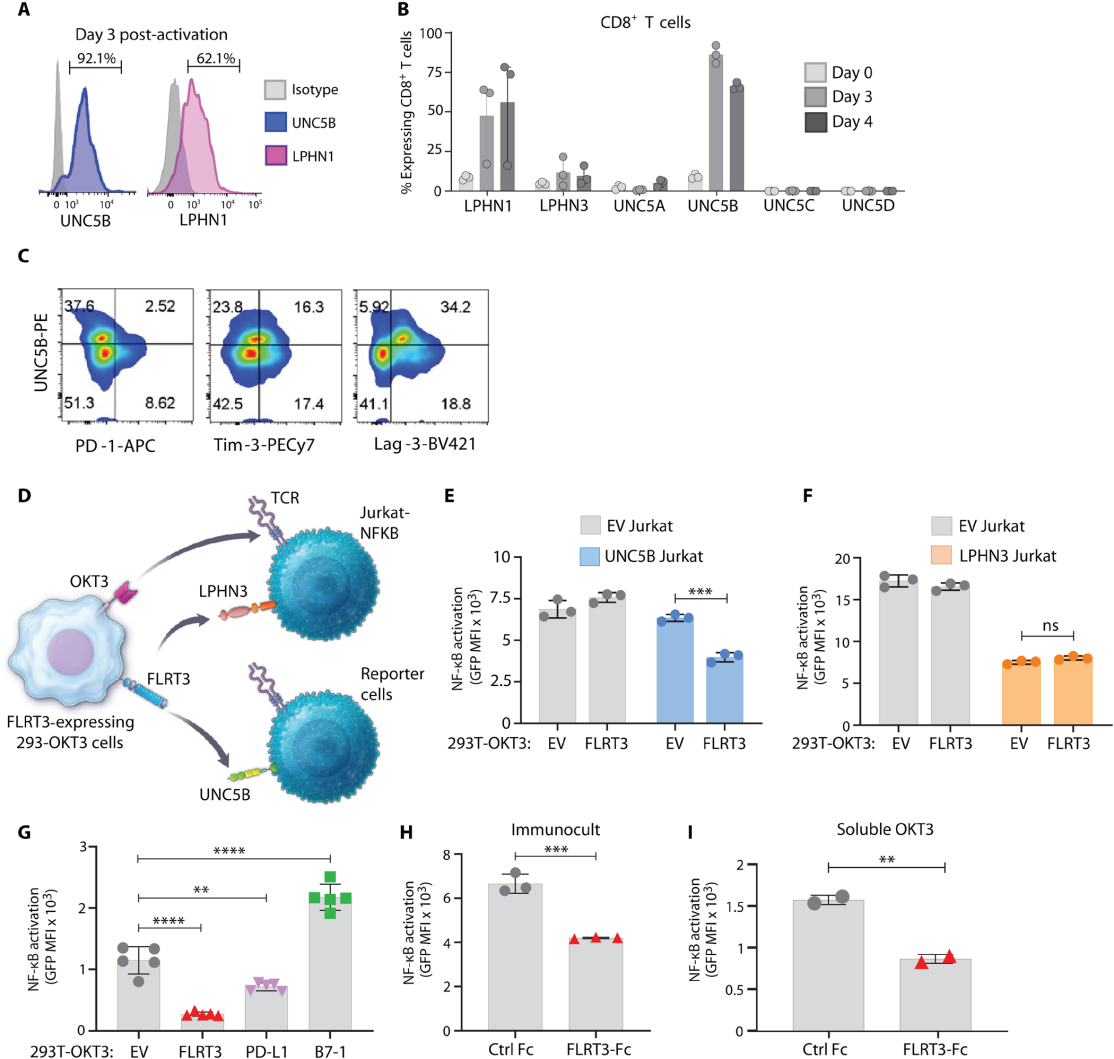
FLRT3 inhibits T cells by interacting with UNC5B receptor. (**A** and **B**) Human PBMCs from three healthy donors were activated with CD3 + CD28 Abs, and FLRT3 binding partner expression was analyzed on CD8^+^ T cells on days 0 (naïve), 3, and 4 (activated). (A) Representative histograms for UNC5B and LPHN1 staining on day 3, and (B) quantification of % CD8^+^ T cell expression. Each dot represents one donor, and error bars denote SD. (**C**) Representative dot plots of UNC5B and checkpoint receptor expression in CD8^+^ T cells from PBMCs activated with CD3 Abs for 10 days. (**D**) Illustration showing Jurkat-NG reporter system. (**E** and **F**) Jurkat-NG cells were transduced with LPHN3 or UNC5B and cocultured with EV- or FLRT3-expressing 293T-OKT3 cells for 16 hours, followed by GFP quantification by flow cytometry. Quantification of GFP MFI in Jurkat-NG cells with or without (E) UNC5B or (F) LPHN3 overexpression. (**G**) UNC5B–Jurkat-NG cells were cocultured with 293T-OKT3 cells expressing FLRT3, PD-L1, or CD80 for 16 hours, and NF-κB activity (GFP) was measured by flow cytometry. Quantification of GFP is shown. (**H** and **I**) UNC5B–Jurkat-NG cells were activated with (H) CD3 + CD28 Abs or (I) soluble CD3 Abs on control Fc of FLRT3 Fc–coated plates for 16 hours, and NF-κB activity (GFP) was measured by flow cytometry. Quantification of GFP levels is shown. For (E) to (I), each data point represents one technical replicate and error bars denote +SD. Data representative of at least two independent experiments. Statistical significance was determined for (E), (F), (H), and (I) by unpaired *t* test and for (G) by one-way ANOVA with Tukey’s post hoc test for multiple comparisons. For all the data, *P < 0.05, **P < 0.01, ***P < 0.001, ****P < 0.0001.

FLRT3 is reported to interact with two families of proteins: uncoordinated 5 (UNC5), Ig domain–containing receptors consisting of UNC5A to UNC5D (*21*, *28*), and latrophilin (LPHN), G protein–coupled receptors consisting of LPHN1 to LPHN3 (*29*, *30*). To study the ligand-receptor pair interactions of FLRT3, we determined the expression of UNCs and LPHNs on T cells. UNC5B and LPHN1 were prominently expressed or up-regulated upon activation and highly expressed on most activated CD8^+^ T cells by day 3 after stimulation (Fig. 3, A and B, and fig. S4). LPHN1 was also expressed on unstimulated CD4^+^ T cells (~90%) but not on CD8^+^ T cells (~8%) (fig. S4A). LPHNs and UNC5s were also variably expressed on B cells and monocytes (fig. S4, A and B). CD8^+^ and CD4^+^ T cells stimulated with Immunocult beads (anti-CD3/28), 293T-OKT3 cells, or OKT3 up-regulated UNC5B after activation (fig. S4, C to F). When coexpression of UNC5B and other checkpoint receptors was evaluated on 10 day activated T cells, UNC5B was primarily coexpressed with LAG-3 in comparison to TIM-3 and PD-1 (Fig. 3C and fig. S4G). Together, these data indicated that FLRT3 receptors are expressed on activated T cells.

Receptors on T cells that were important for FLRT3 coinhibition were evaluated using Jurkat T cells carrying a nuclear factor κB (NF-κB)–green fluorescent protein (GFP) reporter (Jurkat-NG cells) (Fig. 3D). Jurkat-NG cells expressed endogenous LPHN3 but not UNC5B on the cell surface (fig. S5). UNC5B and LPHN3 genes were transduced into the Jurkat-NG cells (fig. S5). Jurkat-NG cell lines were cocultured with 293T-OKT3 empty vector (EV) control or 293T-OKT3-FLRT3^+^ cells to induce T cell receptor (TCR)–CD3ζ signaling in the presence or absence of FLRT3 signaling, followed by analysis of NF-κB–GFP levels by flow cytometry (Fig. 3D). While FLRT3 significantly attenuated NF-κB activity in UNC5B–Jurkat-NG cells, it did not have any effect in EV or LPHN3^hi^–Jurkat-NG cells (Fig. 3, E and F). This indicated that UNC5B is a coinhibitory receptor for FLRT3. LPHN3 transduction itself resulted in decreased NF-κB activation that was independent of FLRT3, while the role of LPHN1 could not be ascertained from this assay system. The diminished NF-κB activity observed by LPHN3 could potentially be the result of its coupling to Gαs and the downstream adenosine 3′,5′-monophosphate (cAMP) pathway following FLRT3-independent/constitutive activation (*31*, *32*).

To compare the inhibition driven by FLRT3-UNC5B with known T cell–suppressive axis PD-1–PD-L1, Jurkat-NG–UNC5B^+^ cells with 293T-OKT3 cells that overexpressed FLRT3, PD-L1, or B7-1 (stimulatory control) were tested. FLRT3-UNC5B inhibition of TCR-driven NF-κB activity was comparable to PD-1–PD-L1 (Fig. 3G). Further, upon TCR stimulation of Jurkat-NG–UNC5B^+^ cells by two different methods, anti-CD3/CD28 Immunocult beads, and soluble OKT3 in the presence of coated FLRT3 Fc protein, we observed specific FLRT3-mediated suppression of NF-κB activity (Fig. 3, H and I). Collectively, these data support FLRT3-mediated coinhibition of T cells through interaction with UNC5B.

### FLRT3 was expressed on tumors and suppressed T cell–based immunotherapies

**Fig. 4.**
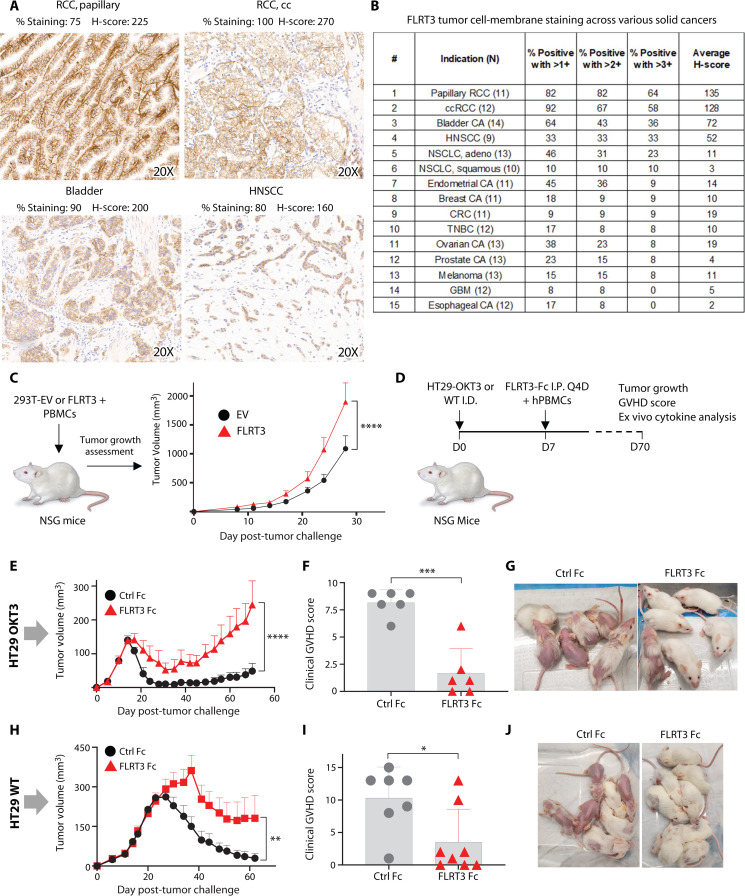
FLRT3 is expressed on tumors and suppressed T cell–based immunotherapies. Tissues from 15 tumor types were stained with an FLRT3 mAb to evaluate FLRT3 membrane expression. (**A**) Representative FLRT3 staining in renal cancer (papillary and clear cell), bladder, and head and neck cancers. (**B**) Pathologist scoring of membrane FLRT3 expression in several tumor types. *N* indicates number of patients screened for a given tumor type. (**C**) FLRT3^+^ renal cancer model in NSG mice. EV- or FLRT3-transduced 293T cells were admixed with human PBMCs and injected in mice intradermally, followed by tumor growth measurements every 2 to 3 days. *n* = 6 animals per group. Data representative of two independent experiments. (**D** to **J**) Soluble FLRT3 Fc testing in HT29-OKT3– or WT-bearing NSG mice. (D) Schematic of experimental design. HT29-OKT3 or WT cells were injected. For the HT29-OKT3 model, PBMCs from a single donor were injected. For the HT29 WT model, PBMCs from five donors were injected. ID, intradermally; IP, intraperitoneally. (E and H) Tumor growth curve. (F and I) Clinical GVHD score measurement on day 59. (G and J) Representative photographs of GVHD development in control Fc– and FLRT3 Fc–treated mice for HT29-OKT3 (E to G) and WT (H to J) models. For the OKT3 model, *n* = 6 animals for control Fc and *n* = 7 animals for FLRT3-Fc. For WT, *n* = 7 animals for control Fc and *n* = 8 animals for FLRT3-Fc. groups. Data representative of two independent experiments. Error bars denote +SEM. Statistical significance was determined for (C), (E), and (H) by paired *t* test and for (F) and (I) by unpaired *t* test. *P < 0.05, **P < 0.01, ***P < 0.001, ****P < 0.0001.

To interrogate the relevance of FLRT3 in human cancers, we determined protein cell surface membrane expression on cancer cell lines and cancer patient formalin-fixed, paraffin-embedded (FFPE) tissues. Membrane FLRT3 was endogenously expressed on several human tumor cell lines (fig. S6). Immunohistochemical (IHC) analysis of cancer FFPE tissues using an FLRT3 monoclonal antibody (mAb) showed the highest membrane FLRT3 expression on papillary renal cell cancer (RCC) and clear cell RCC (Fig. 4, A and B), with less expression on other tumor types (Fig. 4B).

While FLRT3 is expressed in the mouse, we were unable to detect substantial UNC5B expression on activated mouse T cell subsets (fig. S7). This may explain in part why FLRT3 coinhibition of T cells has not been previously identified. Hence, mechanistic and therapeutic studies of FLRT3 were restricted to human cells and humanized animal models.

To model FLRT3-expressing cancer in a cell line–derived xenograft (CDX) tumor model, we intradermally implanted 293T cells that ectopically expressed FLRT3 in NOD.Cg-Prkdc scid Il2rg tm1Wjl/SzJ (NSG) mice with adoptively transferred human PBMCs. FLRT3 overexpression enhanced 293T tumor growth in this model (Fig. 4C).

To further delineate immune function of FLRT3 in vivo, we performed a CDX tumor and graft-versus-host disease (GVHD) model with adoptively transferred human PBMCs and determined whether FLRT3 Fc administration regulated human T cell immune responses against both human tumor cells (allogeneic mismatch) and mouse tissues (xenogeneic mismatch).

The effect of soluble FLRT3 Fc was tested in two HT-29 colon cancer models with either artificial/OKT3 or physiologic/allogeneic activation of T cells. The first model used HT29 cells stably transduced with OKT3 scFv to polyclonally stimulate adoptively transferred human T cells, and the second model consisted of HT29 wild-type (WT) cells with adoptive transfer of mixed, multiple donor PBMCs to induce allogeneic activation of T cells in NSG mice (Fig. 4D). GVHD naturally occurs in these models due to xenogeneic mismatch response of human T cell recognition of mouse major histocompatibility complex (MHC) molecules, and could be exacerbated by OKT3 stimulation or allogeneic activation against tumor cells. FLRT3 Fc inhibited T cell–mediated reduction tumor growth, which was concomitant with attenuation of GVHD in both HT29-OKT3 and WT models (Fig. 4, E to J). As expected, FLRT3 Fc had no impact on HT29-OKT3 tumor growth in the absence of PBMC transfer (fig. S8).

The function of splenic T cells was evaluated at experimental endpoint (d70). While levels of human CD8^+^ and CD4^+^ T cells were comparable between control Fc and FLRT3-Fc groups (fig. S9A), production of IFN-γ and TNF-α (tumor necrosis factor– α) by CD8^+^ T cells was suppressed in the FLRT3-Fc group (fig. S9, B to E). This effect was not observed in CD4^+^ T cells (fig. S9, D and E), suggesting differential role of this pathway in CD8^+^ T cells. Collectively, these data supported an inhibitory function of FLRT3 in soluble form, suggesting additional mechanisms of FLRT3-UNC5B T cell suppression, and potentially additional therapeutic modalities beyond cancer.

**Fig. 5.**
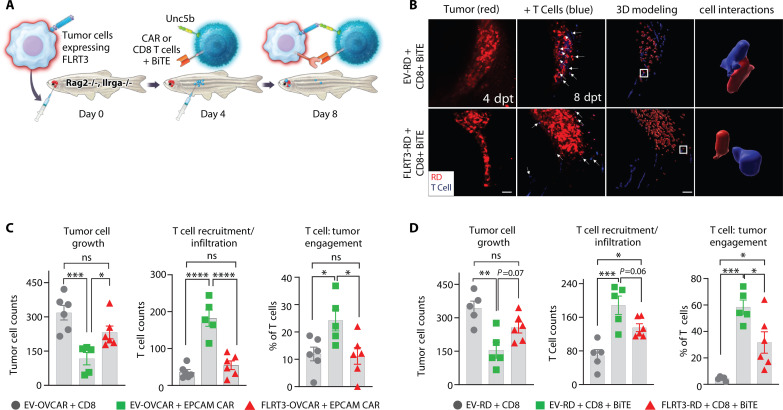
FLRT3 suppresses T cell immunity, infiltration, and tumor engagement in zebrafish models. (**A**) Representative schematic of rag2∆/∆, il2rga−/− zebrafish experimental model to evaluate FLRT3-overexpressing cancer cell lines in the context of CAR-T cells and BiTEs. (**B**) Representative microscopy images showing recruitment (left two panels) and close cell interaction (engagement) (right panel) of T cells (blue) to the RD tumor cells (red). 3D modeling was used to assess T cell:tumor cell engagement. (**C**) OVCAR-5 cells with control CD8^+^ T cells, EV–OVCAR-5 + EpCAM CAR-T cells, and FLRT3–OVCAR-5 + EpCAM CAR-T cell effect on tumor growth (tumor cell counts), T cell recruitment/infiltration (T cell counts), and T cell engagement (percentage of T cells in contact with tumor). (**D**) Same as (C) except using RD tumor model with EV-RD + EGFR-CD3 BiTEs or FLRT3-RD + EGFR-CD3 BiTEs. For (C), six animals for EV + CD8 and FLRT3 + EpCAM CAR groups, and five animals for EV + EpCAM CAR. For (D), five animals for EV + CD8 and EV + CD8 + BiTE groups, and six animals for FLRT3 + CD8 + BiTE. Each data point represents one animal, and error bars denote ±SD. Data representative of two independent experiments. Statistical significance was determined by one-way ANOVA with Tukey’s post hoc test for multiple comparisons. *P < 0.05, **P < 0.01, ***P < 0.001, ****P < 0.0001.

Poor T cell infiltration into solid tumors is a key cause of limited response rates of cancer immunotherapies (*33*). Hence, augmenting the infiltration of T cells to the tumor microenvironment (TME) is essential to improve ICI and T cell–based immunotherapies (*33*, *34*). To further elucidate roles of the FLRT3-UNC5B axis in T cell antitumor immunity and tumor infiltration, we turned to humanized zebrafish models that provide more tractable and convenient way to visualize/measure T cell infiltration/engagement with tumor cells compared to the mouse models (*35*). We used zebrafish xenograft models of ovarian cancer (OVCAR-5) and rhabdomyosarcoma (RD), together with EpCAM Chimeric Antigen Receptor (CAR-T) cells or epidermal growth factor receptor (EGFR)/CD3 bispecific T cell engagers (BiTEs), respectively. Both OVCAR-5 and RD cells showed negligible or no expression of FLRT3 and were transduced to stably express FLRT3 (fig. S10). Both OVCAR-5 and RD cell lines were engineered to also stably express mCherry to assess tumor growth in live animals. In the OVCAR-5 model, EV- or FLRT3-transduced OVCAR-5 cells were engrafted into zebrafish, and 96 hours later, fish were intraperitoneally injected with ViaFluor SE dye–labeled EpCAM–CAR-T cells or control CD8^+^ T cells (Fig. 5A). T cell recruitment, engagement, and tumor growth were assessed 72 hours after T cell injection by confocal imaging (Fig. 5A). In the EGFR^+^ RD model, CD8^+^ T cells were injected with or without EGFR/CD3 BiTEs (Fig. 5, A and B). In the EpCAM/OVCAR-5 model, FLRT3 expression on tumors significantly reduced T cell–mediated antitumor immunity, inhibited T cell TME recruitment/infiltration, and reduced T cell engagement (close interaction) with tumor cells (Fig. 5C). In the EGFR/RD model, similar effects were observed on tumor growth and T cell recruitment but did not reach statistical significance (Fig. 5D), which may be attributed to different T cell mode activity that will require further studies. Overall, these data indicate that the FLRT3-UNC5B axis could suppress CAR-T therapy, as well as BiTE therapies designed to promote antitumor T cell immunity, potentially through inhibition of T cell infiltration to the TME, and reduced engagement of T cells with tumor cells.

### Blockade of FLRT3-UNC5B coinhibition elicited T cell immunity

FLRT3 mAbs that specifically blocked FLRT3 binding to UNC5B or LPHN3 were generated for mechanistic and therapeutic evaluation of the FLRT3 pathway. Anti-human FLRT3 recombinant mAbs on an IgG4P Fc backbone (reduced ability to interact with Fc receptor and complement) that bound specifically to FLRT3 in comparison to FLRT1 and FLRT2 were selected (fig. S11, A to D). FLRT3 Fc bound to both UNC5B Fc and LPHN3 Fc in enzyme-linked immunosorbent assay (ELISA) assays (fig. S11E) and clone NP591 specifically blocked binding of FLRT3 to UNC5B (fig. S11, F and H to J), but not LPHN3 (fig. S11G). FLRT3 clone NP592 specifically blocked binding to LPHN3 but not UNC5B (fig. S11, F and G). Further, NP591 effectively blocked FLRT3 binding to all UNC5 proteins UNC5A to UNC5D (fig. S12).

**Fig. 6.**
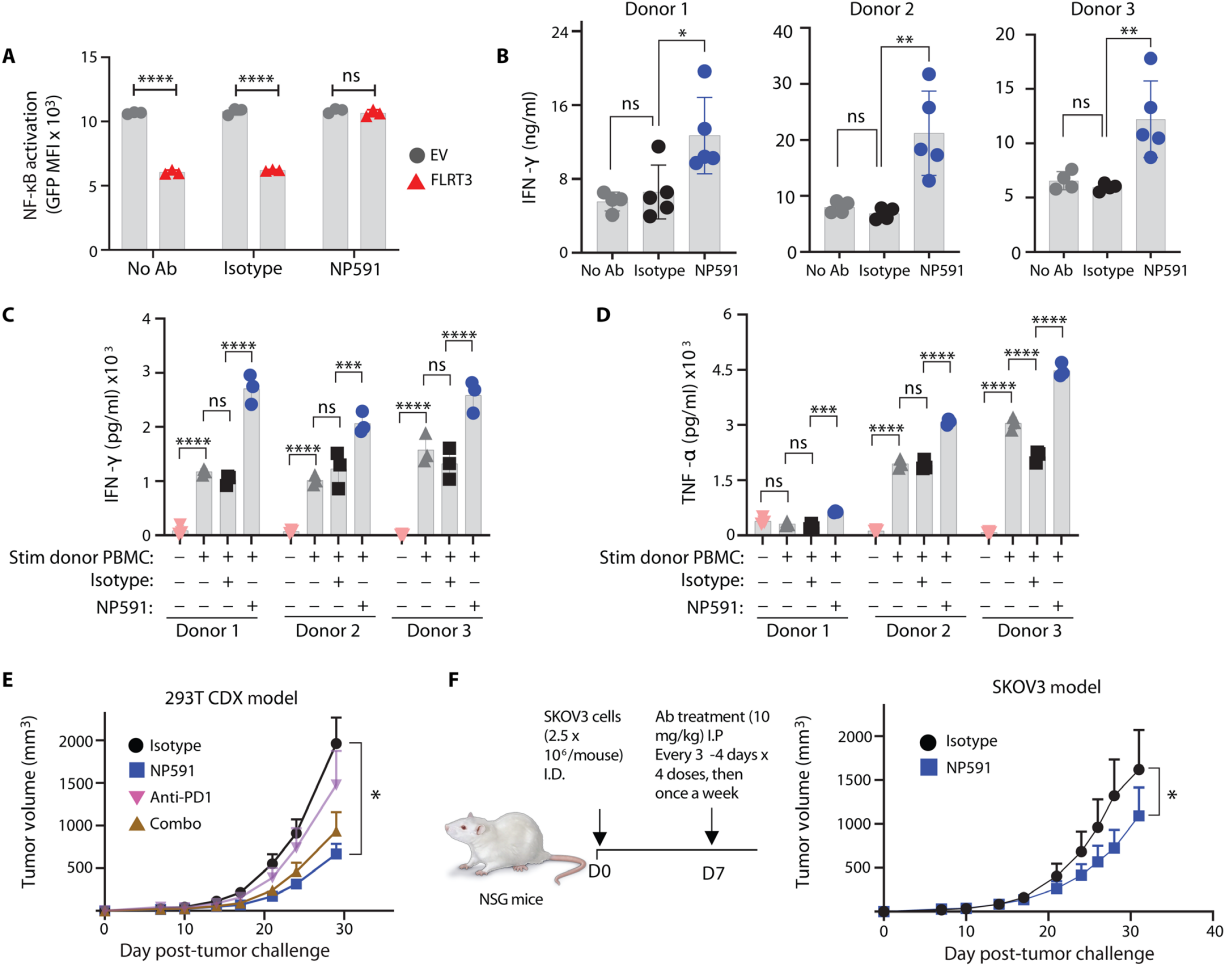
FLRT3-UNC5B blockade promoted T cell function and antitumor immunity. (**A**) Quantification of GFP–NF-κB in UNC5B-Jurkat-NG cells following 16 hours of coculture with EV or FLRT3-293T-OKT3 cells, and NP591 or isotype. Data representative of two experiments. (**B**) Four-day activated human PBMCs from three donors were cocultured with SKOV3 cells with CD3 + CD28 Abs in the presence of NP591 or isotype Ab. Three days later, IFN-γ was measured by ELISA. (**C** and **D**) PBMCs from three healthy donors (responders) were cocultured with donor PBMCs (stimulators) at a 1:1 ratio for 7 days in the presence of irradiated SKOV3 cells. Isotype control or NP591 was added in the cultures on days 0 and 3. On day 7, supernatants were harvested for IFN-γ (C) and TNF-α (D) analysis by ELISA. Bar graph data points in (A) to (D) represent one replicate, and error bars denote SD. (**E**) FLRT3-293T cells were admixed with human PBMCs and injected in mice intradermally. Mice were treated with NP591 anti–PD-1, NP591 and anti–PD-1 combo, or isotype control intraperitoneally starting on day 7 every 2 to 3 days × 3 weeks, then once per week. *n* = 12 per group. (**F**) Human PBMCs were injected intravenously followed by intradermal injection of SKOV3 cells 1 day later. Mice were treated with NP591 or isotype control intraperitoneally starting on day 7 every 2 to 3 days × 2 weeks, then once per week. *N* = 9 for isotype, *n* = 10 for NP591. Error bars denote SEM in (E) and (F). Data representative of two independent experiments. Statistical significance was determined for (A) by unpaired *t* test, for (B) by one-way ANOVA with Tukey’s post hoc test for multiple comparisons, for (C) and (D) by two-way ANOVA with Tukey’s post hoc test for multiple comparisons, and for (E) and (F) by paired *t* test. *P < 0.05, **P < 0.01, ***P < 0.001, ****P < 0.0001.

To test the functional relevance of FLRT3-UNC5B blockade, UNC5B Jurkat-NG cells were cocultured with 293T-OKT3-FLRT3^+^ or EV cells, similar to Fig. 3E, but with the addition of NP591 and NP592. NP591 reversed FLRT3-induced NF-κB inhibition in UNC5B, but not in EV- or LPHN3-expressing Jurkat-NG cells (Fig. 6A and fig. S13C), whereas NP592 had no effect on UNC5B or LPHN3–Jurkat-NG cells (fig. S13, C and D). These results support FLRT3-mediated coinhibition through UNC5B.

To translate in vitro Jurkat assay results to primary human T cells, previously activated human T cells were restimulated with Immunocult (anti-CD3 + anti-CD28 beads) in the presence of SKOV3 ovarian cancer cells that endogenously express FLRT3 for 3 days with NP591 or isotype control. NP591 augmented production of IFN-γ by T cells from multiple donors (Fig. 6B). Further, to test the impact of FLRT3-UNC5B pathway/blockade in a more physiologically relevant setting, we activated PBMCs from three different donors in mixed lymphocyte reaction (MLR) assay in the presence of SKOV3 and A549 cancer cell lines, which naturally express FLRT3. NP591 augmented IFN-γ and TNF-α production approximately two- to threefold in the presence of SKOV3 cells across all donors (Fig. 6, C and D). In A549-based MLR assays, NP591 effects were more prominent on TNF-α production in comparison to IFN-γ (fig. S14, A and B).

To test FLRT3 blockade in vivo, the 293T-FLRT3 CDX tumor model [PD-L1 negative (*36*)] with adoptively transferred PBMCs was tested with FLRT3 mAb NP591, PD-1 mAb, or both. FLRT3 blockade with NP591 significantly reduced tumor growth (Fig. 6E). PD-1 served as a negative control in the model and was not effective alone or in combination with NP591 (Fig. 6E). NP591 was further tested in a SKOV3-FLRT3^+^ ovarian cancer CDX model with endogenous FLRT3 expression in the presence of adoptively transferred human PBMCs. FLRT3-UNC5B blockade by NP591 significantly reduced tumor growth compared to isotype control (Fig. 6F). The effect of FLRT3-UNC5B blockade on tumor growth was confirmed to be dependent on T cells in both 293T-FLRT3 and SKOV3 models (fig. S15).

### FLRT3 blockade promoted T cell–based immunotherapies

**Fig. 7.**
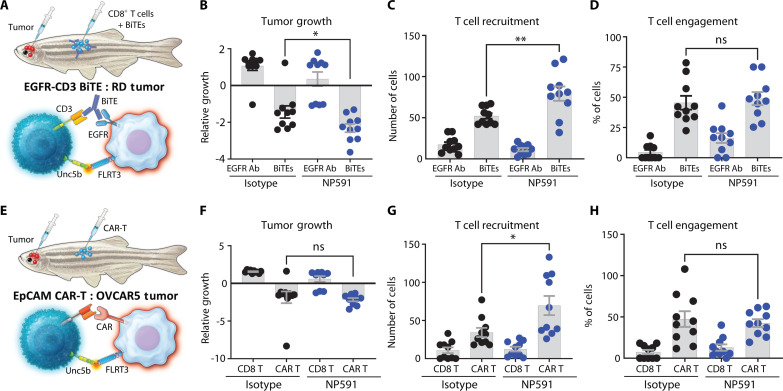
FLRT3-UNC5B blockade promotes T cell–based immunotherapy activity. (**A** to **H**) Zebrafish models were conducted as described in Fig. 5 except with NP591 (10 mg/kg) or isotype control treatments. (A and E) Illustrations for experimental EGFR-CD3 BiTE and EpCAM CAR-T systems, respectively. (B and F) Relative tumor growth, (C and G) T cell recruitment, and (D and H) T cell engagement quantification in RD and OVCAR-5 tumor zebrafish models, respectively. *n* = 10 animals for all the groups. Each data point represents one animal, and error bars denote ±SD. Data representative of two independent experiments. Statistical significance was determined by unpaired *t* test. *P < 0.05, **P < 0.01, ***P < 0.001, ****P < 0.0001.

The therapeutic potential of FLRT3 blockade to enhance T cell–based immunotherapies in solid tumors was tested in zebrafish xenograft models of ovarian cancer (OVCAR-5) and RD together with EpCAM CAR-T cells or EGFR/CD3 BiTEs, respectively (Fig. 7, A and E). NP591 or isotype control mAb was coadministered at the time of CAR-T or CD8^+^ T cell plus BiTE injection in both OVCAR-5 and RD models. Animals were imaged using confocal microscopy 72 hours later for tumor growth, T cell recruitment, and T cell engagement with tumor cells. NP591 significantly reduced tumor growth in the RD, but not in the OVCAR-5 model, likely reflecting the potent antitumor activity of EpCAM CAR-T cells in this experiment, which could only be marginally enhanced by NP591 (Fig. 7, B and F). NP591 promoted T cell recruitment in both models (Fig. 7, C and G), suggesting the role of the FLRT3-UNC5B axis in repulsing T cells from TME. NP591 did not significantly increase T cell engagement in either model (Fig. 7, D and H).

Together, these data identify FLRT3-UNC5B as a checkpoint inhibitor pathway that also inhibits T cell infiltration in tumors (fig. S16). A therapeutic FLRT3 mAb that specifically blocks the FLRT3-UNC5B pathway promoted CD8^+^ T cell antitumor immunity and enhanced CD8^+^ T cell–based immunotherapy in solid tumors.

## DISCUSSION

Immunotherapies that target immune coinhibitory pathways have raised the bar for long-term durable responses to cancer and changed the way we think about oncology care (*2*–*5*). An enduring need is to increase the percentage of cancer patients that are responsive to checkpoint inhibitor therapies (*6*). While there are several avenues to enhance patient responsiveness to checkpoint inhibitors, including modality of treatment, combination therapies, and others, identifying new druggable targets and developable therapeutics to treat nonresponders is essential to drive the field forward (*37*).

AGMs have been well described as playing roles in cancer growth, and there is evidence of immune regulation by axon guidance–related proteins, but limited information exists about their role in receptor-ligand–mediated coinhibition of TCR signaling in cancer (*38*–*44*). Our screening of a cohort of AGMs suggested that many of these proteins may be capable of mimicking coinhibitory ligand-receptor interactions when aberrantly expressed in cancer. The current study focused on the FLRT3-UNC5B pathway, but our screening data implicate AGM coinhibitory mimicry as a potentially broad means of driving immune dysfunction in cancer. Screening and validation of the remaining cohort of AGMs is anticipated to reveal additional immune regulatory pathways for therapeutic intervention. While UNC5B is expressed in mice and important for development, the difference in expression patterns on T cells between mouse and human highlights the importance of screening platforms focused on primary human cells for mechanistic and translational relevance.

Beyond identifying new therapeutic targets, invoking new mechanisms of action beyond those related to current immunotherapies is essential. A major subset of cancer patients are not responsive to checkpoint inhibitors and T cell–based therapies due to absence and exclusion of tumor-infiltrating lymphocytes (TILs), so a major impetus for new immune therapeutics lies in finding ways to enhance infiltration of T cells into the tumor (*33*, *34*, *37*, *45*–*49*). Here, we address these limitations with evaluation of the FLRT3-UNC5B pathway as an inhibitor of not only T cell activation but also T cell infiltration in tumors. Critical for mechanistic understanding and translational development, an FLRT3 blocking Ab specific for blockade of FLRT3-UNC5B interactions enhanced T cell activity in vitro and promoted human T cell–dependent antitumor immunity and T cell recruitment to the TME in vivo.

Expression of FLRT3 on the cell surface of renal cancer suggests that blockade of the FLRT3-UNC5B pathway has the potential to benefit patients that are not effectively treated with existing immunotherapies (*6*, *33*, *34*, *45*–*48*). Future studies will dissect the roles of membrane and soluble FLRT3 on T cell signaling through UNC5B, and downstream functionality, as well as specific roles of this pathway on CD8^+^ and CD4^+^ T cells. Finally, our study exposes AGMs as a new class of checkpoint inhibitors for therapeutic targeting in cancer. Adding to our understanding of the TME expression patterns of AGM pathways will expand our toolbox of immunotherapies, both numerically and mechanistically, ultimately leading to improvements in personalized medicine.

## MATERIALS AND METHODS

### Phylogenetic analysis and TCGA expression analysis

Diversity among 177 genes was evaluated by generating a maximum likelihood tree. The 177 gene IDs were mapped to UniprotKB IDs, and protein sequences were obtained. Each gene is uniquely mapped to Uniprot IDs with maximum sequence length. Multiple sequence alignment is performed using MUSCLE algorithm in MEGA software.

The expression of 177 axon guidance genes from 8431 cancer patients belonging to 34 different cancers was queried using TCGA. Mean expression for each gene was calculated in each of the cancers. A heatmap with two-way hierarchical clustering was generated using gplots v3.1.0 library in R v4.1.0 using heatmap.2 function.

### Cell lines and reagents

HEK293T (293T), HT29, A375, A549, HeLa, and MCF7 cells were purchased from the American Type Culture Collection (ATCC) and cultured in complete Dulbecco’s modified Eagle’s medium (cDMEM). NCI-H446 cells were purchased from ATCC, and Jurkat–NF-κB–GFP cells were purchased from Systems Biosciences (SBI); both were cultured in complete RPMI (cRPMI). SKOV3 cells were purchased from ATCC and cultured in complete McCoy’s 5A medium. RD and OVCAR-5 cell lines were purchased from ATCC and cultured in cDMEM. Complete medium was defined as base medium supplemented with 10% fetal bovine serum (FBS), 1% penicillin-streptomycin, and 1% GlutaMAX (Thermo Fisher Scientific). Leukopaks were purchased from StemExpress or STEMCELL Technologies and used for isolation of PBMCs and T cells. Primary human PBMCs or T cells were cultured in cRPMI. All the cell lines were regularly checked for mycoplasma (InvivoGen) contamination and ensured to be mycoplasma free before conducting studies.

### In-house generated recombinant proteins and Abs

Human FLRT3- and UNC5B-Fc proteins were generated at NextCure by fusing the full extracellular domain of FLRT3 and UNC5B, respectively, with human IgG1 Fc. Human FLRT3-His and UNC5B-His proteins were purchased from Sino Biological. Control Fc protein was a recombinant respiratory syncytial virus (RSV) Ab fused with human IgG1 that was also generated at NextCure using similar methods. To generate anti-FLRT3 Abs, NZB/W mice (The Jackson Laboratory) were immunized with FLRT3-Fc in Freund’s complete adjuvant and boosted with FLRT3-Fc in Freund’s incomplete adjuvant. Fusion, cloning, and subcloning were performed by Precision Antibody (Columbia, MD). Supernatants were screened for binding to FLRT3 by ELISA and cell-based flow cytometry. NP591 and NP592 clones were selected and validated for their strong binding to FLRT3 by plate- and cell-based methods. NP591 and NP592 were synthesized and cloned into human IgG4P (S241P mutant) backbone with reduced ability to interact with Fc receptors and C1Q. Isotype controls for experiments with NP591 and NP592 were recombinant RSV Ab on a human IgG4P backbone that was produced at NextCure using similar methods.

### Flow cytometry

Unless otherwise mentioned, standard flow cytometry staining protocols for cell surface staining were followed to assess protein expression. Briefly, single-cell suspensions of primary cells or cell lines were washed with phosphate-buffered saline (PBS). Thereafter, cells were incubated with appropriate Abs (Table 1) and viability dye was diluted in fluorescence-activated cell sorting (FACS) staining buffer (BioLegend, 420201) at 4°C for 20 to 30 min. In some experiments, cells were incubated with human (BioLegend, 422301) and mouse (BioLegend, 156603) Fc receptor block for 10 min at room temperature before staining with Abs. When secondary staining was necessary, cells were thoroughly washed with PBS after primary Ab incubation step and incubated with corresponding secondary Abs diluted in FACS buffer at 4°C for 20 min. Thereafter, cells were washed again with PBS and either analyzed immediately or fixed in 4% paraformaldehyde (PFA) in PBS for later analysis on flow cytometer.

**Table 1.** List of commercial FACS antibodies/reagents used for cell staining.

Reagent	Vendor	Catalog
Cell Staining Buffer	BioLegend	420201
Zombie Aqua Fixable Viability Kit	BioLegend	423102
Zombie NIR Fixable Viability Kit	BioLegend	423106
CellTrace Violet Cell Proliferation Kit	Invitrogen	C34557
eBioscience CFSE	Invitrogen	65-0850-84
Purified hCD3 antibody (OKT3)	BioLegend	317302
PE-hCD3 antibody	BioLegend	300308
PB-hCD3 antibody	BioLegend	300330
PerCP/Cy5.5-human CD3 antibody	BioLegend	344808
APC/Fire 750-human CD4 antibody	BioLegend	300560
BV711-human CD8 antibody	BioLegend	344734
PE-Cy7 human CD19 antibody	BioLegend	302216
APC-Fire750 hCD14 antibody	BioLegend	367120
BV711-hCD16 antibody	BioLegend	302044
Human UNC5A antibody	Proteintech	20239-1-AP
Human UNC5B antibody	Abcam	ab104871
Human UNC5C antibody	Proteintech	20240-1-AP
Human UNC5D antibody	Proteintech	20241-1-AP
Human LPHN1 pAb	Invitrogen	PA5-77475
Human LPHN3 pAb	Proteintech	20045-1-AP
APC Human PD-1	BioLegend	621610
PE-Cy7 Human Tim-3	BioLegend	119716
BV421 Human Lag-3	BioLegend	369314
Human TruStain FcR blocker	BioLegend	422302
Mouse TruStain FcR blocker	BioLegend	101320
PB-human CD45 antibody	BioLegend	368540
PE-Cy7 mouse CD45 antibody	BioLegend	147703
AF647–anti-mouse IgG Fc	Jackson ImmunoResearch	115-606-071
AF647–anti-human IgG (H+L)	Thermo Fisher Scientific	A21445
PE–anti-rabbit IgG Ab	BioLegend	406421
Rabbit IgG isotype (purified)	BioLegend	910801
Mouse IgG1 isotype (purified)	BioLegend	401402
AF647–anti-rabbit IgG (H+L)	Thermo Fisher Scientific	A21244
AF647–anti-mouse IgG (H+L)	Thermo Fisher Scientific	A21235
PE streptavidin	BioLegend	405204
Commercial human FLRT3 antibody	R&D Systems	AF2795
Anti-goat IgG (H+L) PE-conjugated antibody	R&D Systems	F0107

For all FLRT3 staining, NP591 Ab was used followed by detection using anti-AF647–anti-human immunoglobulin G (IgG) secondary Ab (1:1000). For FLRT3 binding partner assessment, phycoerythrin (PE)–anti-rabbit IgG secondary Ab (1:200) was used.

All the flow cytometry data were acquired on a ZE5 cell analyzer (Bio-Rad) or Attune NxT (Thermo Fisher Scientific) and analyzed on FlowJo software (BD Life Sciences) or FCS Express software (DeNovo). Gating strategies are displayed in fig. S17.

### Gene library screening and validation

293T-OKT3 cells were plated in cDMEM in flat-bottom 96-well plates on day 0. The next day, the cells were transfected with axon guidance–related genes from a Genecopoeia gene set using Lipofectamine 3000 (Thermo Fisher Scientific). On day 3 (48 hours after transfection), transfected 293T cells were irradiated with 3000 J of ultraviolet (UV) radiation. On the same day, freshly thawed frozen PBMCs from healthy human donors were labeled with 5 μM carboxyfluorescein diacetate succinimidyl ester (CFSE) dye (Thermo Fisher Scientific) in cRPMI for 5 min at room temperature, followed by neutralization of excess CFSE and washing steps using cRPMI. CFSE-labeled PBMCs were added on irradiated 293T-OKT3 cells. Cells were cocultured for 72 hours and harvested and incubated with TruStain FcX (BioLegend) for Fc blocking, CD4–allophycocyanin (APC)–eF780 (Thermo Fisher Scientific, 47-0048-42), and CD8-eF450 (Thermo Fisher Scientific, 48-0087-42) in FACS buffer at 4°C. After completion of staining, cells were washed with PBS and resuspended in 3% PFA in PBS and stored at 4°C in the dark until acquisition on a Bio-Rad ZE5 flow cytometer.

Validation assays for gene library screening results were conducted in the same way as described above, except that 293T-OKT3 cells were transfected with new plasmid preparations of FLRT3-, B7-1–, PD-L1–, and EV-expressing plasmids. CFSE proliferation analysis for CD8^+^ and CD4^+^ T cells via flow cytometry and IFN-γ ELISA assay (R&D Systems, DY285) was performed on coculture supernatants.

### Primary T cell assay to evaluate FLRT3 blockade

293T-OKT3-FLRT3 or SKOV3 cells were plated in 96-well plates at 2500 cells per well in cRPMI. The next day, cells were irradiated with 3000 J of UV and freshly thawed human PBMCs were added at 100,000 cells per well in cRPMI supplemented with interleukin-2 (IL-2; 10 ng/ml). In the SKOV3 group, soluble OKT3 (10 ng/ml) was added to activate T cells, and NP591 (10 μg/ml) or isotype control was also added into the coculture. Four days later, supernatants from coculture were collected and subjected to IFN-γ ELISA.

For activated T cell assays, human PBMCs were thawed and activated with soluble OKT3 (50 ng/ml) in the presence of IL-2 (10 ng/ml) for 4 days. Activated T cells were then cocultured with SKOV3 cells restimulated with Immunocult (2.5 μl/ml) for 3 days in the presence of NP591 (10 μg/ml) or isotype control (no IL-2). At the endpoint, IFN-γ levels in the supernatants were measured by ELISA. Recombinant human IL-2 was purchased from PeproTech.

Conventional MLR assays were conducted in the presence of two endogenous FLRT3-expressing cancer cell lines SKOV3 and A549. SKOV3 or A549 cells were plated at 500,000 cells per well in 24-well plates. The next day (day 0), PBMCs from three different donors (5 × 10^6^ per well for each donor) were mixed with stimulator donor PBMCs (5 × 10^6^ per well) at a 1:1 ratio and cultured on SKOV3 or A549 cells that were preirradiated with 3000 mJ/cm^2^ in cRPMI. On days 0 and 3, NP097 or NP591 was added in MLR assay cell cultures at 10 μg/ml. On day 7, supernatants were collected and assessed for IFN-γ and TNF-α production by ELISA. TNF-α ELISA was performed as per the manufacturer’s instructions (R&D Systems, DTA00D).

### Cell line generation

Jurkat–NF-κB–GFP (Jurkat-NG) reporter cells were purchased from System Biosciences (SBI), which stably expressed the GFP reporter driven by minimal cytomegalovirus (mCMV) promoter linked upstream to four copies of the NF-κB response elements. To generate UNC5B-expressing Jurkat cells, UNC5Bv1 was subcloned into pCDH-EF1-IRES-Neo lentivector between EF1 promoter and IRES sequence. For LPHN3, prepackaged lentiparticles carrying LPHN3/ADGRL3 gene in pLTC-ADGRL3-IRES-Neo vector were purchased from G&P Biosciences (LTV7046). Jurkat-NG cells were transduced using standard lentiviral spinoculation protocol, and expression of UNC5B and LPHN3 was confirmed via flow cytometry.

293T-OKT3 cells were generated from WT 293T cells by lentiviral transduction of pCDH-EF1-scFvOKT3-CD14-Puro construct. Transduced 293T cells were then selected under puromycin (10 μg/ml), followed by selection of OKT3-expressing clone via limited dilution cloning.

For 293T-OKT3 cell lines used in Jurkat-NG functional assays, pCDH-EF1-GOI-IRES-GFP lentivector carrying FLRT3, PD-L1, or CD80 genes or EV control was transduced in 293T-OKT3 cells to generate corresponding cell lines. GFP^+^ cells were then sorted via flow cytometry to enrich for cells with the highest Gene Of Interest (GOI) expression.

The 293T-FLRT3 cell line used for in vivo tumor modeling was generated by transducing 293T WT cells with pCDH-EF1-FLRT3-IRES-Puro lentivector and always maintained under puromycin selection (10 μg/ml).

### Jurkat functional assays

293T-OKT3 cells were labeled with CellTrace Violet dye (Thermo Fisher Scientific) at 5 μM concentration at room temperature following the manufacturer’s protocol. Labeled 293T-OKT3 cells were then plated in flat-bottom 96-well plates at 2.5 × 10^4^ cells per well. The next day, 293T cells were irradiated with 3000 J of UV, followed by addition of Jurkat-NG cells at 1 × 10^5^ cells per well. Sixteen hours after coculture, cells were stained with Zombie Aqua Viability dye (BioLegend) and Jurkat cells were analyzed by flow cytometry for GFP [Mean Fluorescent Intensity (MFI)] expression and viability. 293T-OKT3 cells were excluded from the analysis by gating out CellTrace Violet–positive cells. Before each assay, Jurkat-NG cells were treated with G418 (5 mg/ml) to maintain expression of UNC5B.

Experiments to study cell death of Jurkat-NG cells were conducted in the same manner as above except incubation of coculture was 24 hours and cells were stained with AF647–annexin V (BioLegend, 640911) and propidium iodide (PI) (BioLegend, 421301) at the endpoint following the manufacturer’s protocol.

In assays that used Fc fusion proteins, 96-well plates were coated with FLRT3 or control Fc proteins in PBS overnight at 4°C. The next day, plates were washed with PBS three times and Jurkat-NG cells or primary T cells were added with either anti-CD3/anti-CD28 Immunocult (5 μl/ml), soluble OKT3 (200 ng/ml), or titrated coated OKT3.

### Mouse in vivo experiments

All the mouse experiments were conducted at NextCure and were in accordance with standards set by the *Guide for Care and Use of Laboratory Animals, Eighth Edition*. All the mouse experiments were performed using 6- to 8-week-old female NSG mice (The Jackson Laboratory). All the mice were acclimatized for at least 7 days after their arrival before experimentation. As described in figures, 293T-FLRT3 or EV cells (1 million per mouse) were admixed with human PBMCs (3 × 10^5^ per mouse) in 50% Matrigel (Corning) and injected in the right flank intradermally. For the SKOV3 model, human PBMCs (2 × 10^6^ per mouse) were adoptively transferred in mice intravenously 1 day before injecting SKOV3 cells (2.5 × 10^6^ per mouse) in PBS in the right flank of the mice intradermally. For all the models, Ab treatments (10 mg/kg) were started 7 days after tumor challenge and administered intraperitoneally every 2 to 3 days for the first four to six doses followed by once a week until the end of experiment. Tumors were measured using calipers by personnel who were blinded to treatment groups. For PD-1 combo experiments, mice were treated with anti–PD-1 nivolumab clone synthesized in-house in human G4P backbone for research-only use. Tumor measurements were started on day 7 and continued every 2 to 3 days until the end of experiment. Tumor volumes were calculated using the formula *L* × *W*^2^ × ^1^/_2_, where *L* is the length and *W* is the width of tumor. Sample size for each experiment is specified in figure legends. Mice that showed poor tumor engraftment due to technical errors or died earlier than experimental endpoint were excluded from the study. Mice that showed a tumor volume of >2000 mm^3^ or severe sickness/morbidity were euthanized as humane endpoint.

### Mouse ex vivo analysis

At the endpoint, animals were euthanized with CO_2_ and sprayed with 70% Isopropyl Alcohol (IPA) to sanitize the skin around the tumors. Tumors were excised and cut manually into smaller 1- to 2-mm pieces. This was followed by digestion of tissues in RPMI containing enzyme D (100 μl/sample), R (50 μl/sample), and A (12.5 μl per mouse) provided in mouse tumor dissociation kit (Miltenyi Biotec, 130-096-730) at 37°C for 40 min at 37°C on an orbital rotator. Digested tumors were passed through 70-μm cell strainer placed on 50-ml conical tube. Tissue clumps were smashed through the strainer using a syringe plunger to recover as many tumor cells as possible. Thereafter, collected cells were washed three times with cRPMI followed by PBS. Dead cells were removed using Dead Cell Removal (STEMCELL Technologies, catalog 17899), if necessary. Extracted tumor cells were stained with appropriate Abs following human and mouse FcR blocking in FACS buffer following the described procedure and analyzed on a ZE5 flow cytometer.

### Zebrafish in vivo tumor modeling

An optically clear adult rag2∆/∆, il2rgα−/− immunocompromised zebrafish that allows long-term, stable engraftment of human T cells and cancer cells was used to assess gene effects on modulating T cell migration/recruitment and tumor cell killing in vivo. Tumor cell lines were engineered to stably express mCherry (546 nm). Cells were transduced by lentiviral transduction to stably express FLRT3 or EV control. Tumor cells (5 × 10^4^) were engrafted into the peri-ocular musculature, an anatomical superficial site that allows for intravital single-cell imaging using confocal microscopy. Following 96 hours of engraftment, fishes were intraperitoneally injected with ViaFluor 405 nm SE cell proliferation stain (Biotium)–labeled control CD8^+^ T cells or target-specific EGFR CAR T cells (5 × 10^5^ cells per animal) and assessed for T cell recruitment, engagement, and tumor growth 72 hours after injection. A second model used was the EGFR^+^ RD cells and EGFR/CD3 BiTEs with CD8^+^ T cells (Fig. 5). In these experiments, RD cells were transplanted into zebrafish mutant along with either EGFR/CD3 BiTE (50 μg/kg) or EGFR control Ab along with CD8^+^ T cells at 96 hours after engraftment. As above, T cells were labeled ex vivo with ViaFluor SE cell proliferation stain before injection. Three-dimensional (3D) volumetric modeling and distance transformation, distance between spot to surface, and spot close to surface XTension functions in Imaris V10 were used to visualize and accurately quantify differences in engagement between T cells and tumor cells. Tumor cell number could be read out by single-cell imaging of mCherry expression. For experiments testing mAb blockade, anti-FLRT3 NP591 (10 μg/g) or control isotype Ab was coadministered with treatment or control group at 96 hours after tumor engraftment.

### IHC assay

A validated FLRT3 mAb (Santa Cruz Biotechnology, clone A-3, catalog no. sc-514482) was used in the IHC procedure developed by Discovery Life Sciences (DLS; Newtown, PA), and outlined in Table 2 to stain FFPE human tissues from DLS tissue bank, and stained by DLS. Tissue sections were dewaxed using the Leica dewax protocol on the BOND III platform with Leica’s BOND Dewax solution. Antigen retrieval was performed after tissue sections were dewaxed using the Leica BOND III platform. For tissue pretreatment, slides were heated to 100°C for 20 min in high-pH (tris-EDTA, pH 9) Leica BOND ER2 solution. Detection was optimized with inclusion of proteinase K (1:40 dilution) enzyme to further expose the epitopes for binding during tissue pretreatment. The primary Ab was incubated for 1 hour on the Leica BOND III platform, and detection was performed using reagents from BOND Polymer Refine Detection kit from Leica for visualization of mouse primary Abs. Various concentrations of Abs were tested using Reagent Manufacturing Buffer (RMB) Ab diluent with goat serum.

**Table 2.** Procedure for FLRT3 IHC. Leica sequence included intervening washes with BOND wash buffer.

Step	Leica BOND III Reagent
Pretreatment	Samples underwent heat-induced epitope retrieval (HIER) for 20 min using BOND epitope retrieval solution 2 (ER2).
1	Proteinase K—10 min
2	Peroxidase block (Leica Refine)—5 min
3	Primary mAb (clone A-3)—1 hour
4	Post-primary anti-mouse IgG linker—8 min
5	HRP polymer—8 min
6	Mixed DAB refine—10 min
7	Hematoxylin—5 min

### ELISA assays

For ELISA assays evaluating FLRT3 binding to binding partners, 96-well EIA/RIA medium binding ELISA plates (Corning 9017) were coated with appropriate fusion protein (UNC5B-Fc, FLRT3 Fc, LPHN3 Fc, UNC5C Fc, UNC5D Fc, or UNC5A-His) at 5 μg/ml in PBS at 4°C overnight (listed in Table 3). The next day, plates were washed and blocked with 1% bovine serum albumin (BSA) in PBS for 2 hours at room temperature. Plates were washed again, and fusion proteins (5 μg/ml biotinylated FLRT3 Fc or His or 10 μg/ml biotinylated UNC5B Fc or -His) were added in the presence of increasing concentrations of NP591, NP592, or isotype control Abs for 90 min at room temperature (Table 3). In the case of His probe proteins, biotinylated anti-His Ab (1:1000) was incubated for 90 min at room temperature following probe protein incubation step. Plates were then washed, and Eu-streptavidin (1:1000) was added for 20 min at room temperature. Then, 100 μl of enhancement solution was added, following which the fluorescent signals in plates were measured immediately in Envision (PerkinElmer).

**Table 3.** List of binding ELISA-related proteins/antibodies.

Reagent	Vendor	Catalog
Recombinant human UNC5A-His	Sino Biological	13424-H08H
Recombinant human UNC5C Fc	R&D Systems	1005-UN-050
Recombinant human UNC5D Fc	R&D Systems	1429-UN-050
Biotinylated anti-His tag mAb	R&D Systems	BAM050
Recombinant human UNC5B-His	Sino Biological	13606-H08H
Recombinant human FLRT1-His	Sino Biological	11389-H08H
Recombinant human FLRT2-His	Sino Biological	11296-H08H
Recombinant human FLRT3-His	Sino Biological	11166-H08H
Recombinant human LPHN3 Fc	R&D Systems	8915-LN-050
Biotinylated mouse Fc specific Ab	Invitrogen	A16094
Biotinylated rabbit secondary Ab	Abcam	ab6720-1MG

ELISAs assessing NP591’s binding to FLRT1, FLRT2, and FLRT3 proteins were conducted in a similar way wherein NP591 or isotype control Ab was coated at 5 μg/ml and probed with FLRT-His proteins (5 μg/ml).

### Flow cytometry binding assays

NP591 binding to FLRT3-expressing cell lines was tested by incubating NP591 with dissociated 293T-hFLRT3/EV or A549 single-cell suspensions in FACS buffer at increasing concentrations for 1 hour at 4°C. Cells were washed with PBS and incubated with secondary AF647–anti-human IgG (1:1000) and zombie aqua viability dye (1:100) in FACS buffer, followed by PBS wash steps and acquisition of data on flow cytometer.

NP591 effect on FLRT3-UNC5B binding was evaluated by incubating biotinylated FLRT3-Fc (4 μg/ml) with UNC5B/EV–Jurkat-NG cells in 96-well plates (at 2 × 10^5^ cells per well) in 1% BSA in PBS at 4°C for 1 hour. NP591 or isotype control Ab was also added in this mix at increasing concentrations. Then, cells were washed thoroughly with PBS and stained with secondary PE-streptavidin (1:400) and Zombie Aqua viability dye (1:100) in FACS buffer at 4°C for 30 min. This was followed by PBS wash steps, fixation of cells with 4% PFA in PBS, and acquisition of data on a flow cytometer.

### Study design

The sample sizes for all the functional in vitro and in vivo studies were empirically determined based on the expected experimental variability and the statistical power required to run the appropriate statistical tests. PBMCs used in in vitro or in in vivo experiments in this study were isolated from the blood of healthy donors. The objective for mouse in vivo tumor model experiments was to assess differences in the tumor growth between experimental groups, and endpoints were determined depending on the tumor growth rates and whether the collected data points were sufficient to compare the differences in the tumor growth in conclusive manner. Mice that showed poor tumor engraftment due to technical errors or died earlier than experimental endpoint were excluded from the study as predefined criteria. For all mouse studies, tumors were measured using calipers by personnel who were blinded to treatment groups. For zebrafish in vivo models, tumor growth and T cell infiltration/engagement and endpoints were determined based on previously validated experimental design. The technical and experimental replicates for all the experiments are reported in the figure legends. All the animals were randomly assigned to various experimental groups. All the mouse experiments conducted at NextCure were in accordance with standards set by the *Guide for Care and Use of Laboratory Animals, Eighth Edition*.

### Statistical analysis

All the statistical analyses were conducted using Excel (Microsoft) or Prism (GraphPad). Statistical significance was determined using unpaired or paired two-tailed Student’s *t* test for experiments comparing two groups, and one- or two-way analysis of variance (ANOVA) with Tukey’s post hoc test for multiple comparisons for experiments comparing more than two groups. To calculate EC_50_ (median effective concentration) for FLRT3 binding, maximal binding (100%) was defined as the highest average MFI value (binding) observed across all the data points and the rest of the data points were normalized accordingly. Following logarithmic transformation of Ab concentrations, nonlinear regression fit analysis of normalized dose response was conducted in Prism to determine the EC_50_ values. Percent change in proliferation in Fig. 2C and fig. S3D was calculated using the following equation

$$\%\;\text{Change in proliferation}=\frac{\%\;\text{proliferating cells in}\;X-\%\;\text{proliferating cells in}\;\text{EV}}{\%\;\text{proliferating cells in EV}}\times 100$$

where *X* = FLRT3/PD-1/B7-1.

Percent change in IFN-γ in Fig. 2E was calculated using the following equation

$$\%\;\text{Change in IFN}-\gamma =\frac{\text{IFN}-\gamma \;\text{levels in}\;X-\text{IFN}-\gamma \;\text{levels in}\;\text{EV}}{\text{IFN}-\gamma \;\text{levels in}\;\text{EV}}\times 100$$

where *X* = FLRT3/PD-1/B7-1.
