# Supplementary material for: The FLRT3-UNC5B checkpoint pathway inhibits T cell–based cancer immunotherapies
Source: Sci Adv. 2024 Mar 1;10(9):eadj4698. doi: 10.1126/sciadv.adj4698 (PMC10906930; doi:10.1126/sciadv.adj4698)
Supplement: Supplementary file 1 — Figs. S1 to S17 Table S1 [file sciadv.adj4698_sm.pdf]

Science Advances
AAAS

Supplementary Materials for

The FLRT3-UNC5B checkpoint pathway inhibits T cell–based cancer immunotherapies

Kushal Prajapati *et al.*

Corresponding author: Dallas B. Flies, fliesd@nextcure.com

Sci. Adv. **10**, eadj4698 (2024)
DOI: 10.1126/sciadv.adj4698

This PDF file includes:

Figs. S1 to S17
Table S1

Supplemental Figure 1

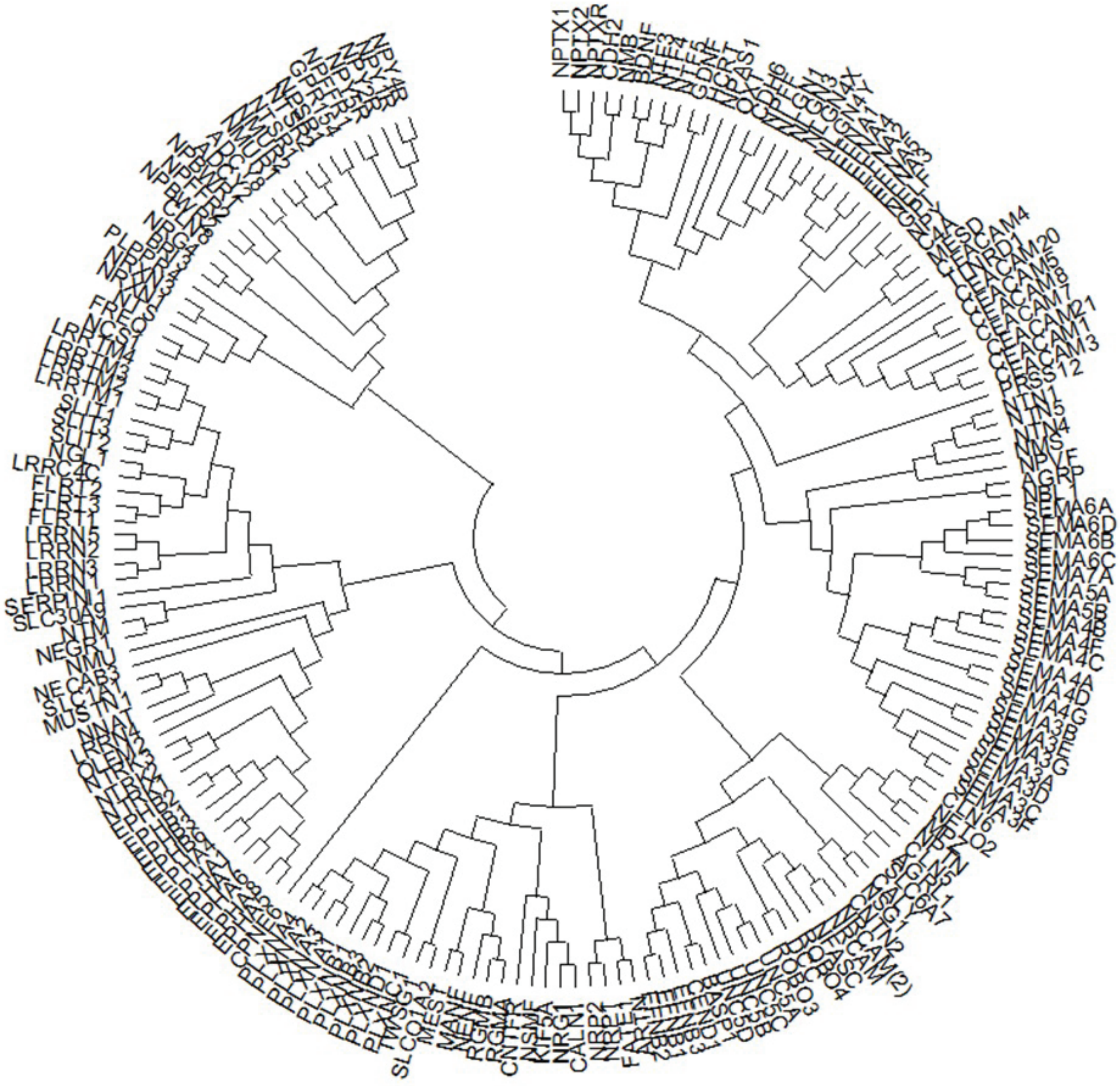

Fig. S1: Phylogenetic map of axon guidance-like genes. Genes with axon guidance related activities were identified by literature and keyword searches. Identified genes were assessed based on phylogeny and displayed in a circular phylogenetic map.

Supplemental Figure 2

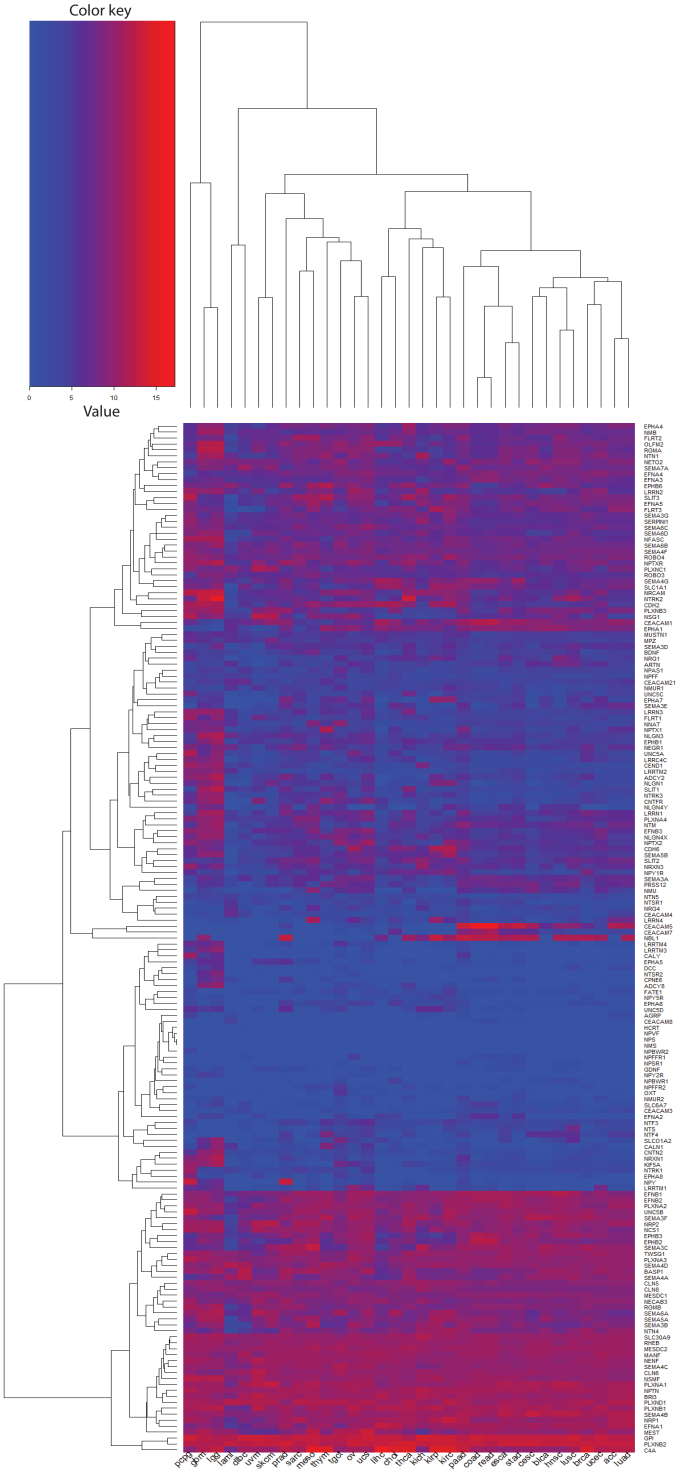

Fig. S2: Axon guidance molecule expression in cancer. Genes with axon guidance related activities were identified by literature and keyword searches. The expression of 177 axon guidance genes from 8431 cancer patients belonging to 34 different cancers were queried using TCGA. Mean expression for each gene were calculated in each of the cancers. A heatmap with two-way hierarchical clustering was generated using gplots v3.1.0 library in R v4.1.0 using heatmap.2 function.

Supplemental Figure 3

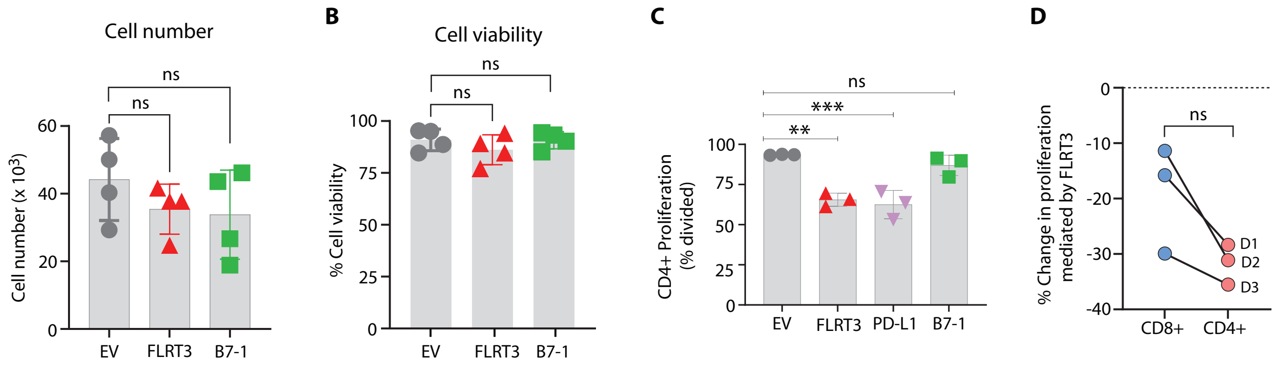

Fig. S3: Effect of FLRT3 transfection on proliferation and viability of 293T cells in vitro. (**A** and **B**) 293T-OKT3 cells were transiently transfected with EV, FLRT3 and B7-1 genes and 6 days later (**A**) cell number and (**B**) % viability were determined by trypan blue method. (**C**) CFSE labelled human PBMCs from 3 healthy donors were co-cultured with 293T-OKT3 cells transfected with FLRT3, PD-L1 and B7-1 genes followed by analysis of proliferation by CFSE dilution. Quantification of proliferating CD4+ T cells is shown for one representative donor. (**D**) Summary data quantifying % change in proliferation in FLRT3 group compared to EV control for CD8+ and CD4+ T cells separately for 3 donors. For (**A** to **C**) statistical significance was determined by One-way ANOVA with Tukey's post-hoc test for multiple comparisons. For (**D**) statistical significance was determined by pairwise student's T test. *p<0.05, **p<0.01, ***p<0.001, ****p<0.0001

Supplemental Figure 4

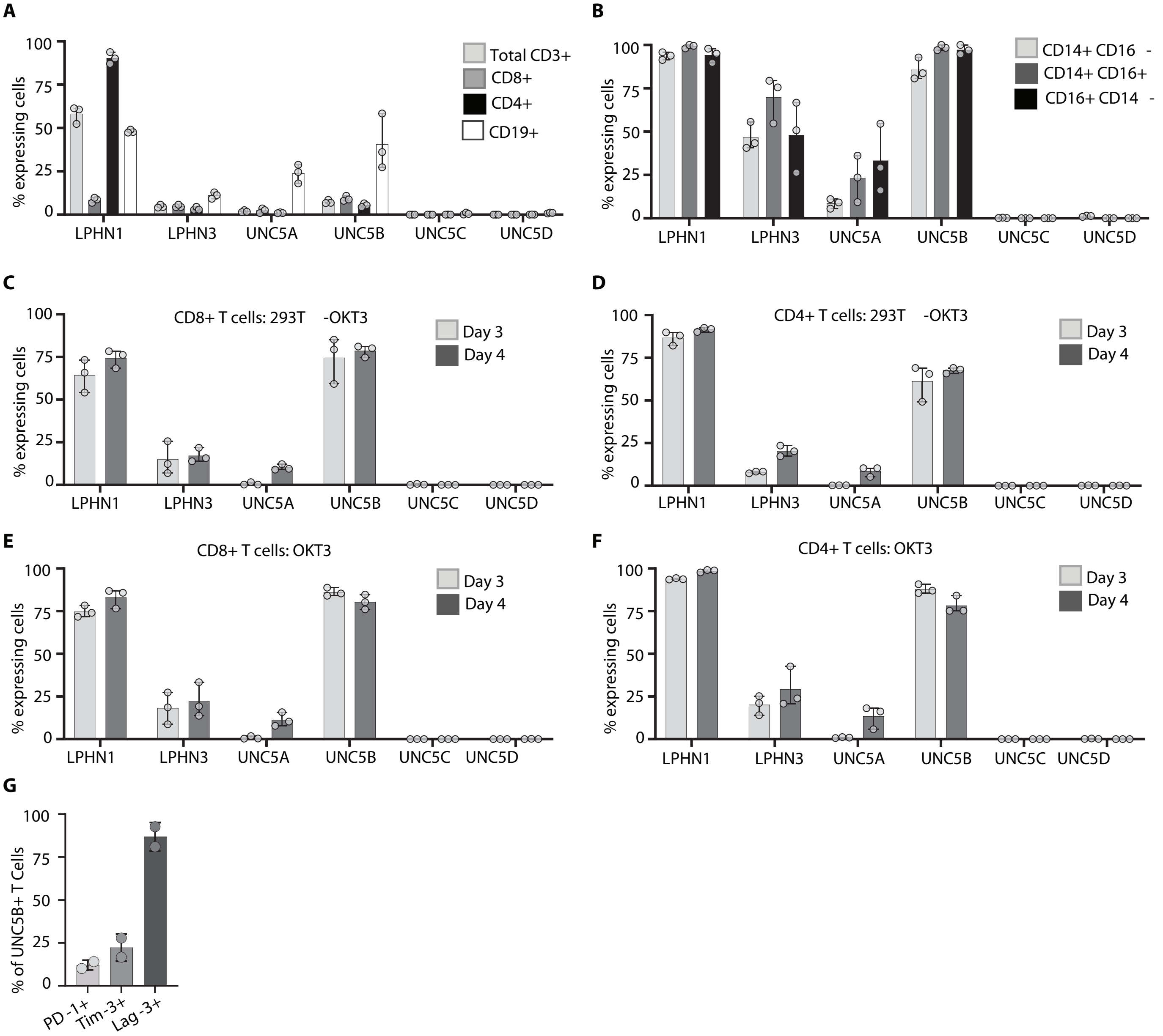

Fig. S4: UNC5B is expressed on activated human T cells. (**A** and **B**) Human PBMCs from 3 healthy donors were analyzed for expression of FLRT3's binding partners LPHN1, LPHN3, UNC5A-D on B and T cells (**A**) monocytes (**B**) on day 0. (**C** to **F**) Human PBMCs from 3 healthy donors were activated by co-culturing with 293T-OKT3 (**C** and **D**) or with soluble OKT3 (**E** and **F**) and expression of FLRT3's binding partners LPHN1, LPHN3, UNC5A-D was measured on T cells on day 3 and 4 (activated). CD8+ (**C** and **E**) and CD4+ T cells (**D** and **F**) data are shown separately. (**G**) Human PBMCs from 2 healthy donors were activated with soluble OKT3 for 10 days and UNC5B expression was analyzed along with other checkpoint receptors PD-1, TIM-3, LAG-3. Quantification of PD-1+, TIM-3+, LAG-3+ cells within the UNC5B+ population is shown. Each data point represents one donor and error bars denote ± Range in bar graphs.

Supplemental Figure 5

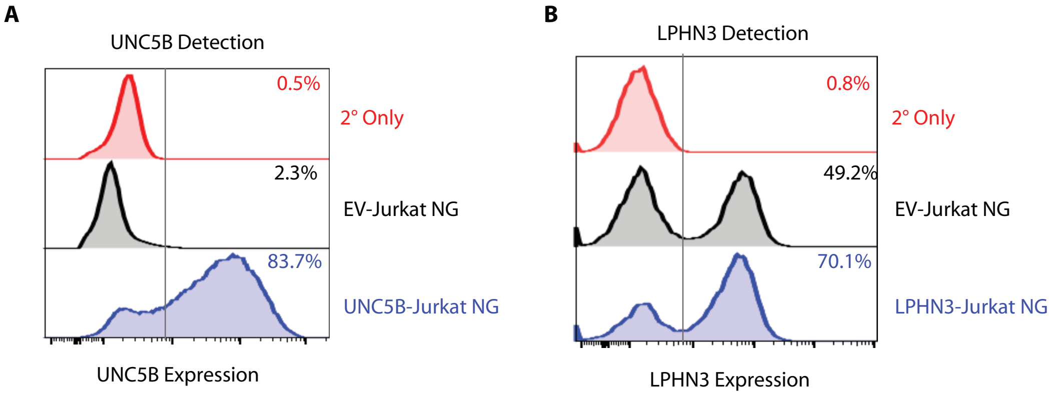

Fig. S5: Expression of UNC5B and LPHN3 on transduced Jurkat-NG cells. (**A** and **B**) Jurkat-NG cells were transduced with LPHN3 or UNC5B and expression levels of UNC5B (**A**) and LPHN3 (**B**) on transduced Jurkat-NG cell lines were analyzed by flow cytometry.

–

Supplemental Figure 6

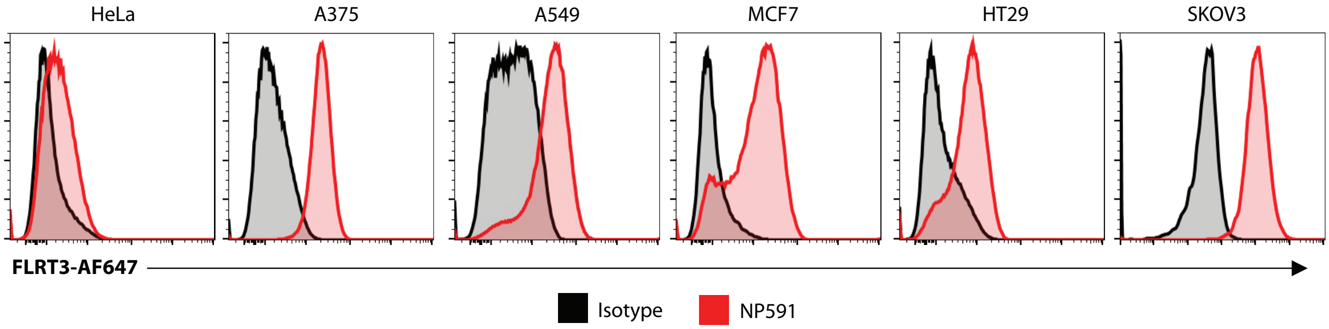

Fig. S6: FLRT3 expression on cancer cell lines. Indicated tumor cell lines were stained using FLRT3 mAb and analyzed by flow cytometry for FLRT3 expression.

Supplemental Figure 7

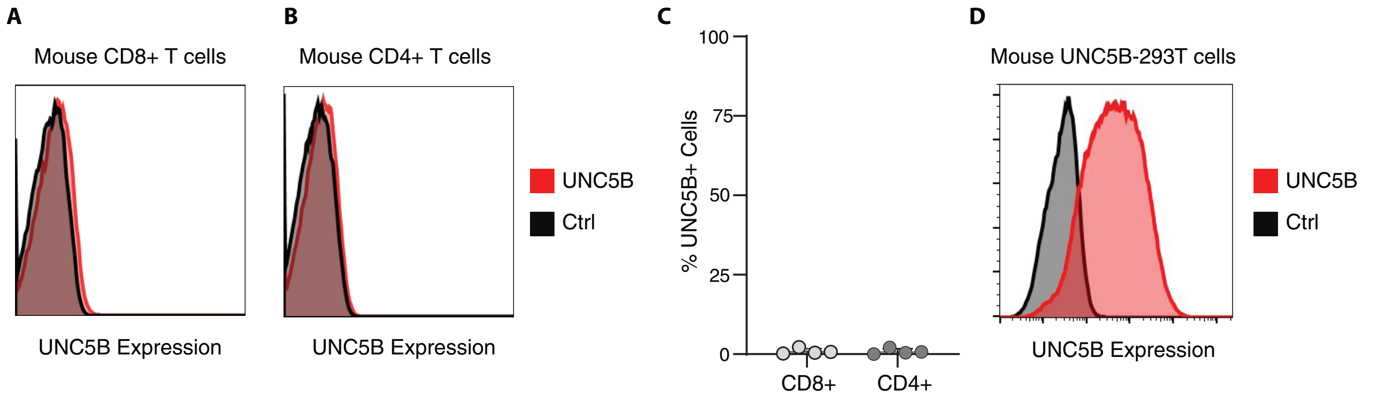

Fig. S7: UNC5B is not substantially expressed on mouse T cells. (**A** to **C**) Mouse CD3+ T cells were isolated from spleens of Balb/c mice and activated with mouse dynabeads and IL-2 for 4 days followed by assessment of UNC5B expression on CD8+ (**A**) and CD4+ (**B**) T cells by flow cytometry. (**C**) Quantification of % UNC5B+ cells on activated mouse T cells from n = 4 mice. Each data point represents one animal and error bars denote ± SD. (**D**) Cross reactivity of UNC5B Ab to mouse UNC5B was confirmed by staining mouse UNC5B-transfected 293T cells via flow cytometry.

Supplemental Figure 8

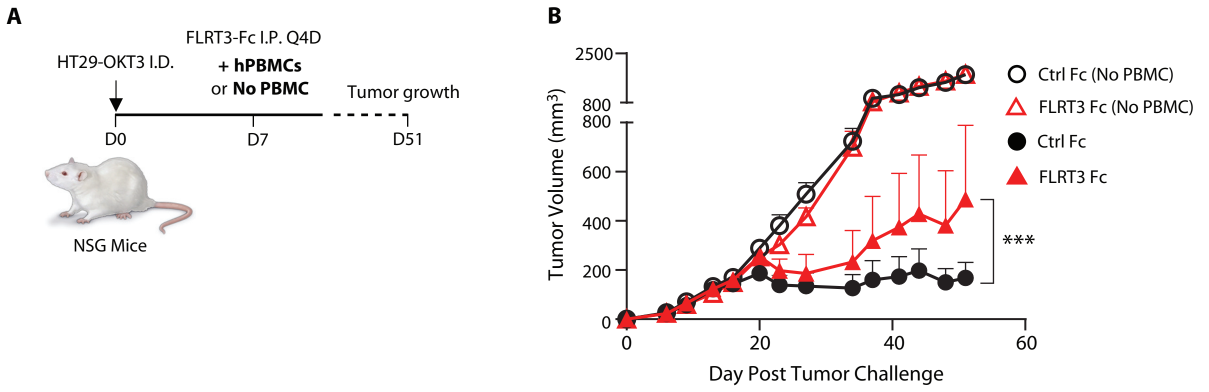

Fig. S8: FLRT3 Fc mediated suppression of tumor growth in dependent on PBMC/T cell activity. Soluble FLRT3-Fc testing in HT29-OKT3 bearing NSG mice with or without PBMC administration. (**A**) Schematic of experimental design. (**B**) Tumor growth curve. n = 9 animals for No PBMC groups and 8 for groups wherein PBMCs were injected. Error bars denote + SEM. Data are representative of two independent experiments. *** $p<0.001$ by paired T-test.

Supplemental Figure 9

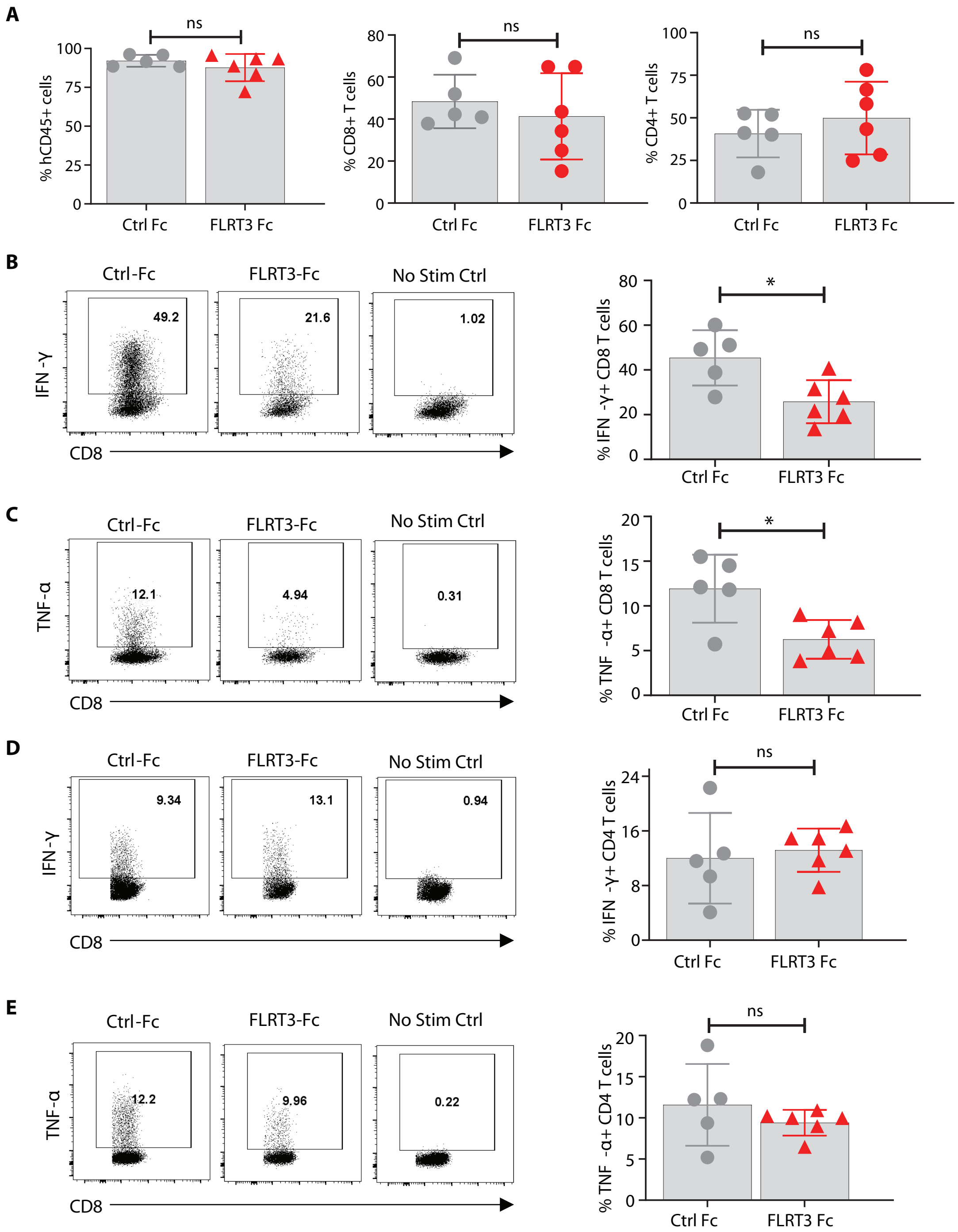

Fig. S9: FLRT3 suppresses inflammatory cytokine production in T cells. Soluble FLRT3-Fc testing in HT29-OKT3 bearing NSG mice was performed as described in Fig. 4C-F. (**A**) Levels of hCD45+, CD8+ and CD4+ T cells found in the spleen of mice treated with Ctrl or FLRT3-Fc at the endpoint. (**B** to **E**) Spleens from mice in this model were collected at endpoint and re-stimulated with coated OKT3 and brefeldin A overnight and cytokine production was evaluated the next day by intra-cellular flow cytometry. Representative flow charts and quantification of IFN-γ (B) and TNF-α (**C**) production in CD8 T cells and IFN-γ (**D**) and TNF-α (**E**) production CD4 T cells. For b-e, Each data point represents one mouse and error bars denote + SD in bar graphs. Data representative of at least two independent experiments For all the data, * p<0.05, ** p<0.01, *** p<0.001, *** p<0.0001 by unpaired T-test.

Supplemental Figure 10

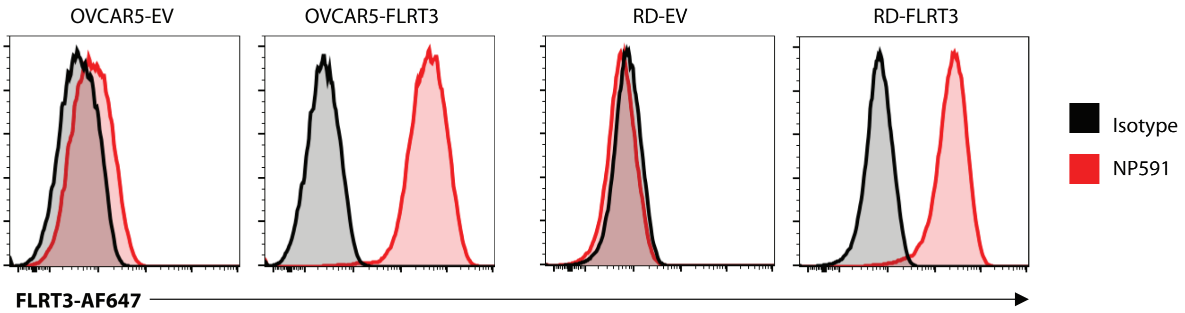

Fig. S10: FLRT3 expression on cancer cell lines. Indicated tumor cell lines were stained using FLRT3 mAb and analyzed by flow cytometry for FLRT3 expression.

Supplemental Figure 11

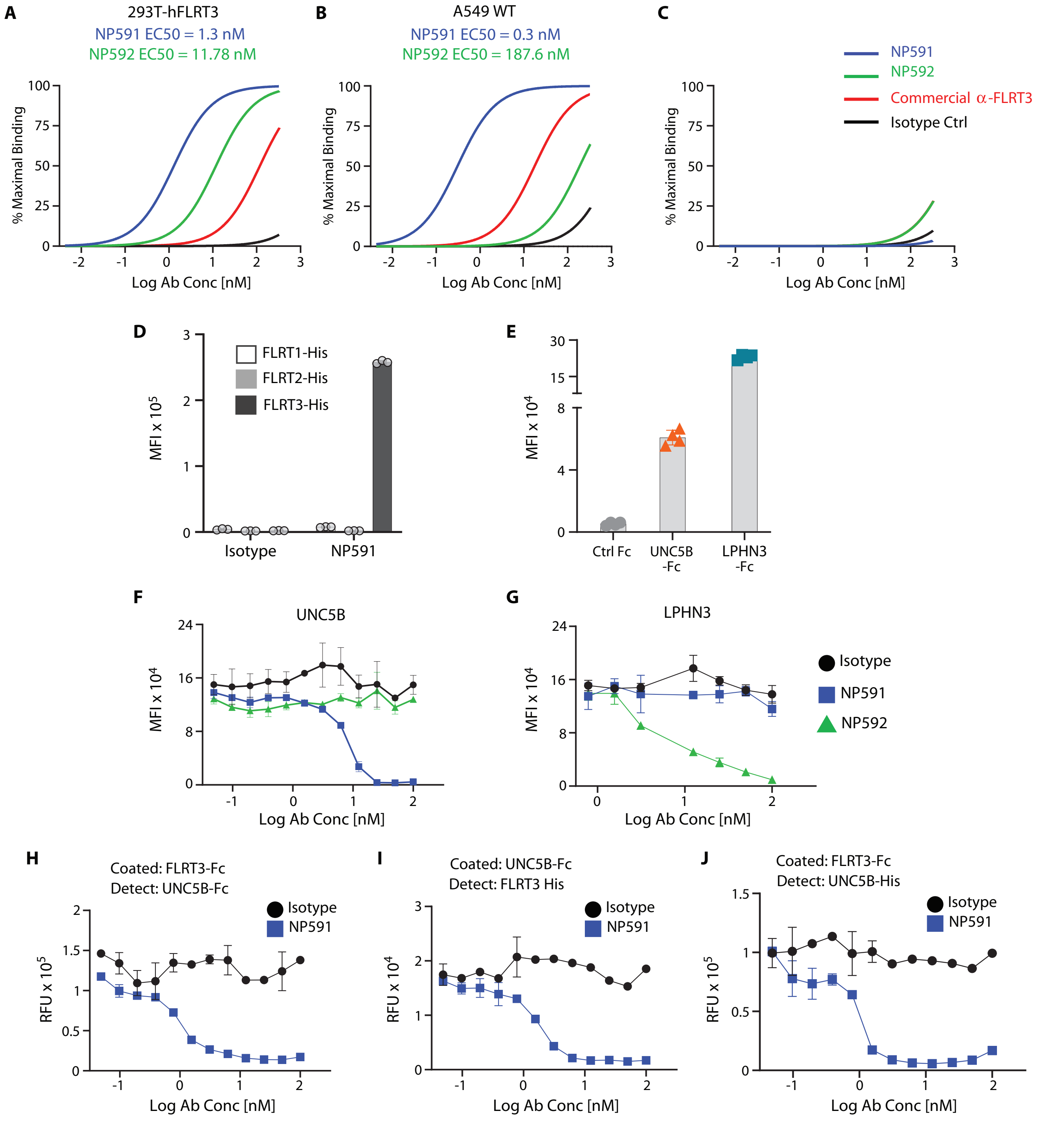

Fig. S11: FLRT3 mAb specifically binds to FLRT3 and blocks FLRT3-UNC5B interaction. (**A** to **C**) Binding of FLRT3 mAbs to human FLRT3 over-expressing 293T cells (**A**), endogenous FLRT3 expressing A549 cell line (**B**), and 293T-EV (**C**) cells. Binding was measured with serial fold dilution via flow cytometry. EC50 values were calculated using nonlinear regression model in Graphpad Prism. (**D**) Binding of FLRT3 mAb to coated FLRT1, FLRT2 and FLRT3-His proteins in ELISA. (E to G) Testing effect of FLRT3 mAb on UNC5 and LPHN-FLRT3 interactions in ELISA. (**E**) Binding of FLRT3-Fc to coated UNC5B and LPHN3-Fc proteins in ELISA. Binding of UNC5B-Fc (**F**) and LPHN3-Fc (**G**) to coated FLRT3-Fc was determined in the presence of increasing conc of FLRT3 mAb. (**H** to **J**) Binding of UNC5B-Fc to coated FLRT3-Fc (**H**), FLRT3 His to coated UNC5B-Fc (**I**) and UNC5B-His to coated FLRT3-Fc (**J**) was determined in the presence of increasing conc of FLRT3 mAb in ELISA. All data representative of at least two independent experiments.

Supplemental Figure 12

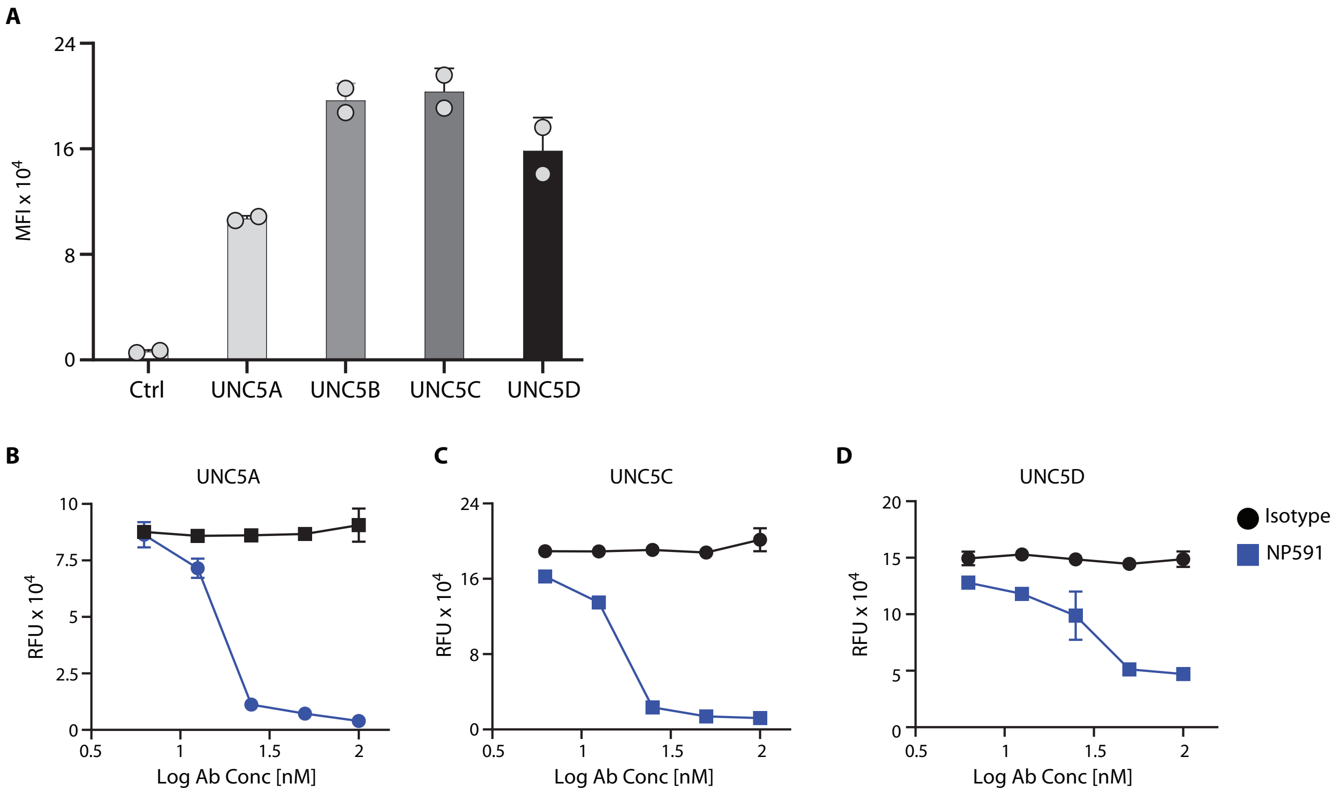

Fig. S12: FLRT3 mAb blocks FLRT3 interaction with all UNC5 receptors. (**A**) Binding of FLRT3-Fc to coated UNC5A-D proteins in ELISA. (**B** to **D**) Binding of FLRT3-Fc to coated UNC5A-His (**B**), UNC5C-Fc (**C**), and UNC5D-Fc (**D**) was determined in the presence of increasing conc of FLRT3 mAb in ELISA. Each datapoint represents one technical replicate in all bar graphs. Error bars denote ± SD in all the error bars.

Supplemental Figure 13

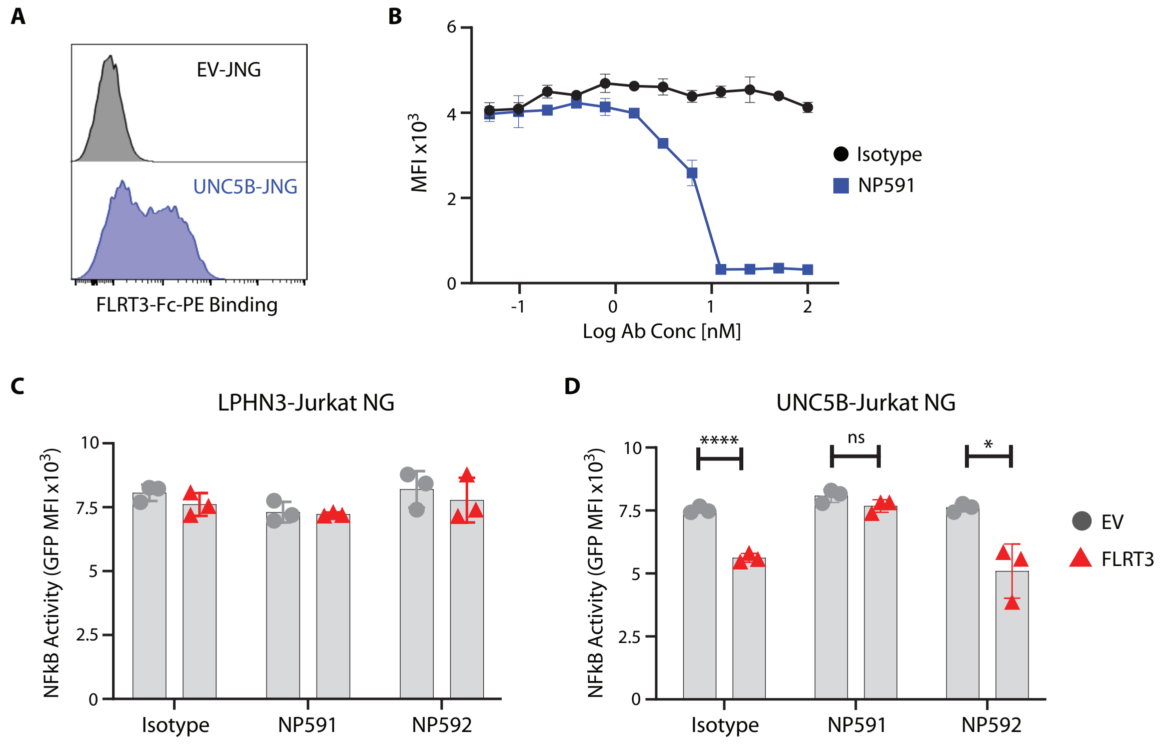

Fig. S13: FLRT3 mAb blocks FLRT3 interaction UNC5B in cell-based assays. (A and B) Testing effect of FLRT3 mAb on FLRT3-UNC5B interaction via flow cytometry. (A) Binding of FLRT3-Fc to EV or UNC5B-Jurkat NG cells. (B) Binding of FLRT3-Fc to UNC5B-Jurkat NG cells in presence of increasing conc of NP591. (C) Quantification of GFP expression (indicative of NFkB activity) in LPHN3-Jurkat NG cells following 16 hours co-culture with EV or FLRT3-293T-OKT3 cells with 10 ug/mL FLRT3 mAbs NP591 and NP592 by flow cytometry. (D) Same as (C) except using UNC5B-Jurkat NG cells. Each datapoint represents one technical replicate in all bar graphs. Error bars denote ± SD in all the error bars. * $p<0.05$, ** $p<0.01$, *** $p<0.001$, **** $p<0.0001$ by unpaired T-test.

Supplemental Figure 14

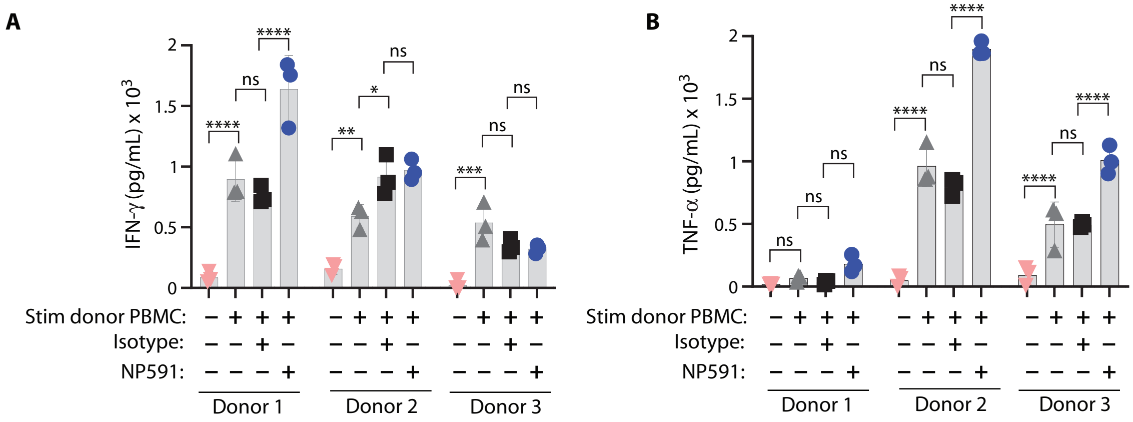

Fig. S14: FLRT3 blockade enhances IFN-γ and TNF-α production by PBMCs in MLR assay. (**A** and **B**) PBMCs from 3 healthy donors (responders) were cocultured with a stimulatory donor PBMCs at 1:1 ratio to induce allogeneic response for 7 days in the presence of irradiated, endogenous FLRT3 expressing A549 lung cancer cells. Isotype control or NP591 antibodies were added in the cultures at 10 ug/mL on day 0 and day 3. On day 7, supernatants from the MLR assay cultures were collected and evaluated for the production of IFN-γ (**A**) and TNF-α (**B**) by ELISA. Each datapoint represents one technical replicate in all the bar graphs for each donor. Error bars denote ± SD. * $p<0.05$, ** $p<0.01$, *** $p<0.001$, **** $p<0.0001$ by Two-way ANOVA Tukey's post-hoc test for multiple comparisons.

Supplemental Figure 15

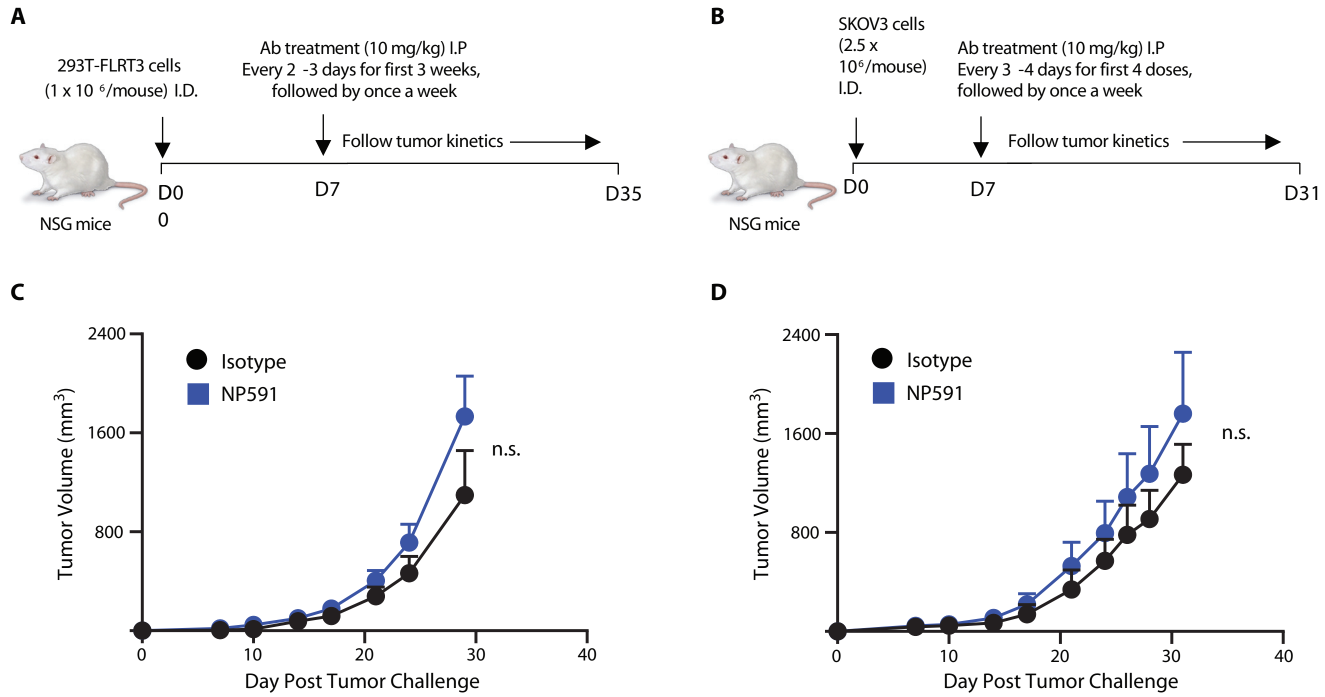

Fig. S15: FLRT3 blockade efficacy in vivo is dependent on human T cells. (**A** and **B**) Schematic of experimental designs of (**A**) 293T-FLRT3 and (**B**) SKOV3 models without human PBMC administration. (C and D) Tumor growth curves from 293T-FLRT3 model (**C**) and SKOV3 model (**D**). Error bars denote + SEM in (**C** and **D**) All the data are representative of two independent experiments.

Supplemental Figure 16

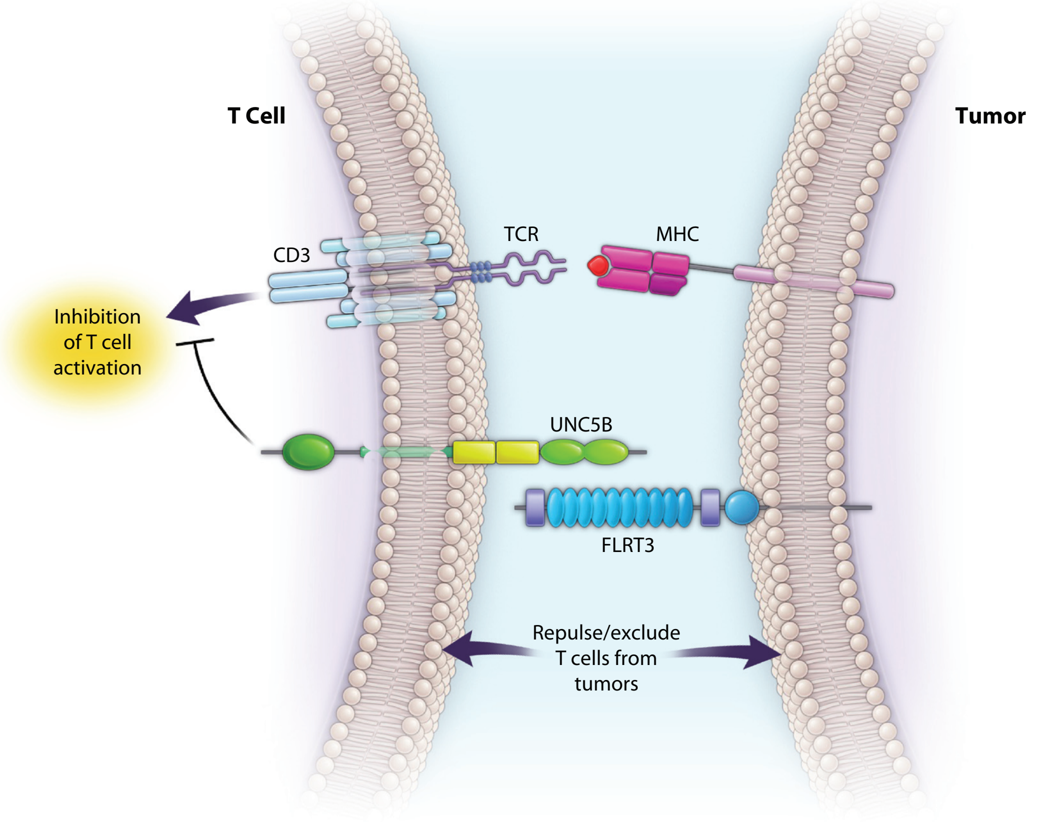

Fig. S16: Working model of FLRT3-UNC5B mediated immune co-inhibitory mimicry. FLRT3 expression on tumor cells in cancer functions as a mimic of traditional coinhibitory ligands when it binds to UNC5B that is aberrantly upregulated on Ag-stimulated T cells. UNC5B signaling is capable of inhibiting TCR-CD3 signaling that prevents T cell activation and recruitment and/or infiltration to the tumor microenvironments.

Supplemental Figure 17

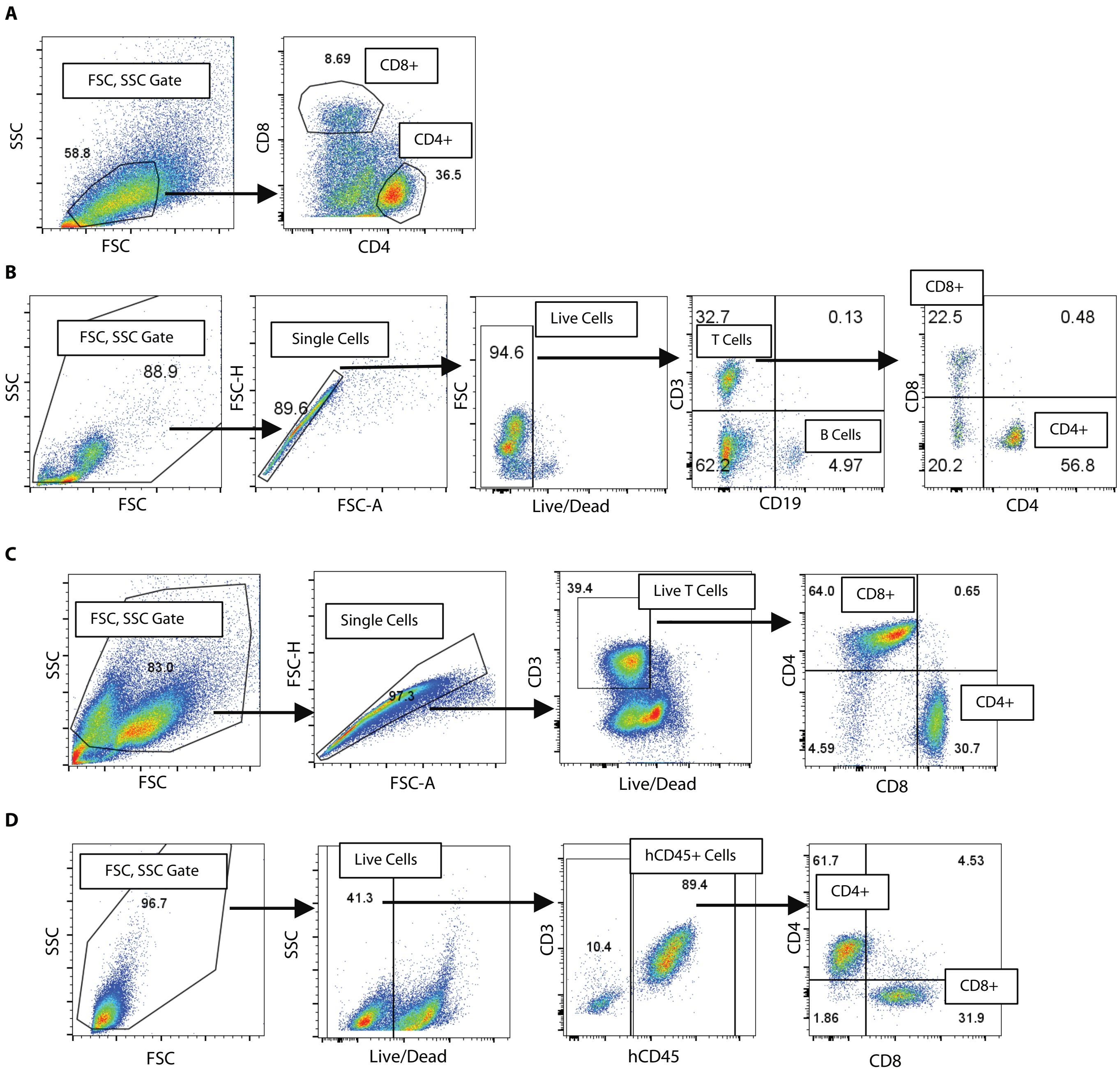

Fig. S17: Gating strategies. Gating strategies used to evaluate CD8+ and CD4+ T cell for proliferation in AGM screening (**A**), assessment of FLRT3 binding partner expression (**B**), assessment of UNC5B, PD-1, TIM-3 and LAG-3 (**C**) and ex vivo analysis from the spleens of Ctrl Fc or FLRT3-Fc treated mice (**D**).

Supplemental Table 1

Gene ID	CD8 Rank-1	CD8 Rank-2	CD8 Rank-3	CD8 Rank-4	CD8 Rank Score	CD4 Rank-1	CD4 Rank-2	CD4 Rank-3	CD4 Rank-4	CD4 Rank Score
ADGRL1-v2	52	8	12	7	16	43	4	8	8	13
B7-1	3	50	1	16	11	1	1	1	2	1
DCC	31	44	19	31	30	38	27	27	44	34
EFNA1	13	1	13	13	4	19	3	4	13	6
EFNA2	53	47	35	57	55	51	28	39	56	49
EFNA3	49	48	56	54	56	35	49	48	50	51
EFNA4-v1	16	24	7	6	7	16	44	14	3	17
EFNA5	23	37	46	28	35	12	40	47	29	29
EFNB1	38	42	45	25	45	52	48	40	15	43
EFNB2	47	45	17	35	40	31	47	37	49	45
EFNB3	54	49	11	23	36	44	43	23	28	35
EPHA1	30	51	51	46	50	33	52	52	47	53
EPHA2-v1	25	39	48	22	34	32	34	25	17	23
EPHA3-v1	28	40	41	39	43	28	45	36	34	37
EPHA4-v2	17	38	40	21	28	39	36	44	21	36
EPHA5-v1	36	34	38	38	42	42	38	43	51	48
EPHA6-v1	18	17	24	16	13	15	22	29	19	20
EPHA7-v1	26	26	33	41	31	41	39	33	35	41
EPHA8-v1	55	18	18	4	21	55	14	7	5	18
EPHB1	32	56	31	42	49	37	56	32	43	46
EPHB2-v1	56	29	23	32	38	56	19	41	40	44
EPHB2-v2	41	41	34	43	48	21	42	34	39	33
EPHB4	29	55	54	48	53	29	55	54	54	55
EPHB6	27	53	57	51	54	25	54	57	53	54
FLRT2-v1	46	27	14	50	37	45	30	30	41	40
FLRT3-v1	57	52	52	53	57	57	51	53	52	57
NRG1	8	5	26	11	6	14	11	22	11	10
NRG1-vGGF2	14	21	25	2	8	17	26	38	12	21
NRG1-vHRGg	6	9	4	12	3	3	5	6	14	3
NRP2	34	15	16	47	26	33	21	19	45	27
NRP2-v1	50	43	15	49	47	53	35	26	38	42
PD-L1-v1	39	57	55	28	51	36	57	56	32	50
PLXNA2	22	30	9	36	22	13	23	9	24	14
PLXNA4-v1	42	12	29	9	19	26	20	28	9	19
PLXNB2	45	32	44	24	41	50	41	49	33	47
PLXNB2-alt	1	2	6	10	1	5	7	5	7	2
PLXNB3-v1	48	54	53	30	52	46	53	55	30	52
PLXNC1-v1	11	3	28	20	9	10	2	13	16	7
PLXND1	24	25	49	27	29	27	18	51	25	28
ROBO3	37	36	30	45	44	48	50	46	55	56
SEMA4A-alt	40	4	5	56	24	2	6	3	48	12
SEMA4A-v1	20	6	2	55	18	18	10	2	46	16
SEMA4B	21	14	42	15	20	20	16	21	18	15
SEMA4C	15	23	36	8	17	24	37	42	10	25
SEMA4D	12	11	47	5	12	23	9	18	6	8
SEMA4G	43	31	37	19	32	49	28	35	20	31
SEMA5A	19	28	43	18	25	30	31	45	27	32
SEMA5B	51	33	3	14	23	54	33	16	26	30
SEMA6A-v2	7	20	50	37	27	9	12	50	37	24
SEMA6D-v1	33	35	39	33	39	22	32	20	42	26
SEMA6D-v4	4	16	22	34	15	7	25	31	31	22
SEMA6D-v6	2	19	20	26	10	8	13	15	23	11
SEMA7A-v1	35	22	21	52	33	47	24	17	57	38
UNC5A	10	13	10	43	14	6	17	12	22	9
UNC5B-v1	5	7	32	3	5	11	8	10	4	5
UNC5D-v2	44	46	27	40	46	40	46	24	36	39
Vector control	9	10	8	1	2	4	15	11	1	4

Table S1. FIND-IO screening results from 4 independent screens of normal, healthy donors. CD8+ and CD4+ T cell proliferation based on percent CFSE divided cells for axon guidance-related proteins transfected and expressed as proteins on 293T-OKT3 cells. 293T-OKT3 cells were UV-irradiated and co-cultured with primary total PBMCs (cryo-recovered for fresh) or primary enriched T cells for 72 hours, followed by assessment of T cell proliferation by flow cytometry. Proliferation values were converted to a rank from 1 to 57 for each screen and then averaged for a combined rank score for CD8+ and CD4+ T cells
